# Synergistic Effects of Antioxidant Blends: A Comparative Study on Oxidative Stability of Lipids in Feed Matrices

**DOI:** 10.3390/antiox14080981

**Published:** 2025-08-10

**Authors:** Xuyang Gao, Yong Xiao, Wen Li, Liting Xu, Jianmin Yuan

**Affiliations:** State Key Laboratory of Animal Nutrition and Feeding, College of Animal Science and Technology, China Agricultural University, No. 2 Yuanmingyuan Western Road, Hai Dian District, Beijing 100193, China; yang2022@cau.edu.cn (X.G.); xiaoyong_hengyang@cau.edu.cn (Y.X.); S20243040839@cau.edu.cn (W.L.); s20233102249@cau.edu.cn (L.X.)

**Keywords:** combined antioxidants, single antioxidants, oil oxidation, decay kinetics, evaluation approach

## Abstract

Lipid peroxidation driven by polyunsaturated fatty acid (PUFA) oxidation compromises feed quality and animal health. Single antioxidants (e.g., ethoxyquin (EQ), butylated hydroxytoluene (BHT)) face limitations including dose-dependent toxicity, bioaccumulation risks, and inadequate protection against multistage oxidation. Composite systems leveraging complementary mechanisms offer a promising alternative. This study evaluated synergistic efficacy of rationally formulated composite antioxidants (combining synthetic radical scavengers and metal chelators) versus single-component systems in enhancing lipid oxidative stability in high-fat animal feed. The basal diet containing oxidized oil served as the control group (CON). Seven groups were supplemented with the basal diet as follows: Treatment A, 36 g/ton Butylated Hydroxytoluene (BHT); Treatment B, 60 g/ton Ethoxyquin (EQ); Treatment C, 132 g/ton EQ; Treatment D, 10 g/ton EQ + 12 g/ton BHT; Treatment E, 10 g/ton EQ + 12 g/ton BHT + 6 g/ton Citric acid (CA); Treatment F, 20 g/ton EQ + 6 g/ton BHT + 6 g/ton CA; and treatment G, 2 g/ton EQ + 25 g/ton BHT + 6 g/ton CA. Oxidative stability was assessed over a 10-week period under natural storage (T0-T10) and acute thermal stress (120 °C drying for 2 h followed by ambient storage; HT0 to HT10). Oxidative stability was assessed via: antioxidant capacity (DPPH (2,2-Diphenyl-1-picrylhydrazyl)/ABTS (2,2′-Azinobis (3-ethylbenzothiazoline-6-sulfonic acid) scavenging, total antioxidant capacity), physical indices: Color (L*, a*, b*), and chemical oxidation markers: conjugated dienes (CD), peroxide value (PV), p-anisidine value (p-AV), malondialdehyde (MDA), acid value (AV), total oxidation (TOTOX). Superior synergistic performance of the ternary blend (Treatment E) was demonstrated versus singles (A/B/C). Retention of radical scavenging capacity was significantly enhanced, with greater stability observed under accelerated storage. Primary oxidation (PV) and secondary oxidation (MDA, p-AV) were most effectively suppressed by Treatment E. Superior color stability (minimal L* change) was maintained under thermal stress. The lowest TOTOX values were achieved across all conditions by Treatment E. Stage-specific vulnerabilities were shown by single antioxidants (BHT volatilization; pro-oxidative effects of EQ at high doses). Comprehensive, temperature-resilient protection was delivered collectively by the synergistic EQ+BHT+CA system (Treatment E) via combined radical quenching and metal chelation. The inherent limitations of individual antioxidants were effectively overcome by the optimized composite, enabling reduced total dosage while substantially extending the lipid oxidative stability period.

## 1. Introduction

Lipid peroxidation in high-fat plant-derived animal feed, driven by oxidative degradation of polyunsaturated fatty acids (PUFAs), presents a dual challenge to both feed quality and animal health [1]. The oxidation products not only reduce nutritional value, but may also lead to intestinal and hepatic inflammation and cellular metabolic dysfunction in animals [2], affecting skeletal muscle development [3], necessitating robust antioxidant strategies.

Current approaches employ either single-component antioxidant systems or advanced multi-component, which are widely used in the Chinese market. Single-component systems include the following: Ethoxyquin (EQ) exerts its antioxidant effect primarily by stabilizing free radicals through intramolecular resonance delocalization, rendering them inactive and incapable of attacking other fatty acid molecules. This mechanism makes EQ highly effective in suppressing the propagation stage of lipid oxidation [4]; Butylated hydroxytoluene (BHT), which functions as a primary radical scavenger by terminating propagation chains through hydrogen donation [5]. While single antioxidants effectively prolong the induction period and reduce primary oxidation products (peroxide value, PV; conjugated dienes, CD), particularly under thermal stress, their utility is compromised by significant drawbacks, including dose-dependent toxicity risks (e.g., EQ-associated hepatotoxicity [6,7]), potential bioaccumulation, and generation of harmful byproducts during processing [8].

In contrast, composite antioxidant systems address these limitations through complementary mechanisms: combining radical scavengers with redox cyclers enhances electron transfer efficiency [9], while integrating primary antioxidants with metal chelators (citric acid) simultaneously inhibits both radical-mediated and metal-catalyzed oxidation [10,11], synergistic co-antioxidant to regenerate primary antioxidants via hydrogen donation. The metal-chelating capacity of citric acid provides continuous protection by sequestering pro-oxidant ions (Fe^2+^, Cu^2+^), thereby suppressing both initiation (radical formation) and propagation (hydroperoxide decomposition) phases [10]. This dual mechanism provides sustained protection throughout the oxidation process, significantly slowing the rise in both primary and secondary oxidation products.

Comprehensive evaluation of lipid oxidation requires a multi-parametric approach incorporating primary product analysis, such as PV for early-stage hydroperoxides [12], secondary product detection, such as p-anisidine value for unsaturated aldehydes and malondialdehyde (MDA), and integrated indices like total oxidation value for holistic assessment [13,14].

Building on these insights, we hypothesize that rationally designed composite antioxidants—combining synthetic radical scavengers (e.g., EQ, BHT) with metal chelators (e.g., citric acid) based on the antioxidant product formula in the Chinese market—will provide stage-specific protection against lipid oxidation, enhance safety through reduced doses of individual components, and improve efficacy against both primary (PV, CD) and secondary (p-Anisidine Value, MDA) oxidation markers. Such systems are thus expected to overcome conventional antioxidant limitations while maintaining processing stability and feed safety.

## 2. Materials and Methods

### 2.1. Chemical Reagents

Butylated hydroxytoluene (BHT, 99%) and citric acid (CA, 99%) were bought from Aladdin Chemical Co., (Shanghai, China), with ethoxyquin (EQ, 95%) sourced from Sinopharm Chemical Reagent Co., Ltd. (Shanghai, China). Antioxidants were formulated based on the commercial specifications and incorporated into soybean oil via ultrasonication (25–40 kHz) to form stable microemulsions. These microemulsions were uniformly blended into feed matrices through intensive mixing to ensure homogeneity prior to granulation.

### 2.2. Experiment Design, Feed Oxidized Oil Extraction and Antioxidant Samples Preparation

The basal diet was formulated based on Arbor Acres broiler nutrition specification (2022) the composition was listed in Supplement Table S1. A gradient-optimized composite strategy was employed to evaluate dose-dependent and synergistic effects. The basal diet was as the control (CON). Single-component groups established baseline efficacy: Treatment A served as industry-standard phenolic control; Treatment B represented conventional chelator dosage; Treatment C evaluated toxicity thresholds. Binary composite. Treatment D assessed primary antioxidant synergy. Ternary composites featured fixed citric acid with varied radical scavenger-to-chelator ratios: Treatment E targeted optimized regeneration stoichiometry; Treatment F simulated metal-rich scenarios; Treatment G focused on radical-dominated oxidation systems. Basal diet supplemented with 200 g/ton of different antioxidants. The detailed purity and effective content are shown in Table 1.

Feeds were mixed for pellet, and in crumbles. Feed samples from all treatments were stored in room-temperature storage (25 ± 1 °C) and accelerated oxidation (120 ± 1 °C for 2 h followed by room storage). Triplicate samples per group (100 g) were collected at 7-day intervals over 10 weeks (T0–T10, corresponding to weeks 0–10 at normal temperature, HT0–HT10, corresponding to weeks 0–10 at high temperature). Liquid oil was extracted from feed matrices using a validated protocol [15] for lipid oxidation analysis.

Parallel standalone antioxidant mixtures (matching feed-supplemented concentrations) were prepared without feed incorporation and subjected to identical storage conditions and synchronous sampling (1 g per group). This dual-track approach enabled comparative evaluation of antioxidant retention rates, free radical scavenging capacity, and degradation kinetics between feed-embedded and isolated systems under naturally and thermally accelerated oxidization.

### 2.3. DPPH (2,2-Diphenyl-1-picrylhydrazyl) Degradation Efficiency, ABTS (2,2′-Azinobis(3-ethylbenzothiazoline-6-sulfonic acid) Radical Absorbing Capacity and Total Antioxidant Capacity Assay

The antioxidant capacity analyses were conducted using three commercial assay kits according to standardized protocols. The DPPH Free Radical Scavenging Capacity Assay Kit (BC4750) and ABTS Free Radical Scavenging Capacity Assay Kit (BC4770) were obtained from Solarbio Science & Technology Co., Ltd. (Beijing, China). The Total Antioxidant Capacity Assay Kit (S0116) was acquired from Beyotime Institute of Biotechnology (Shanghai, China). All experimental procedures were strictly performed in accordance with the manufacturer’s protocols provided in each kit’s technical manual.

### 2.4. Determination of Color Parameters (L*, a*, b*)

The color parameters (L, a*, b*) of the samples were measured using a chroma meter (Konica Minolta Sensing, Inc., Tokyo, Japan, CR-410; CIE Lab color system) calibrated with a standard white calibration plate (Y = 93.5, x = 0.3134, y = 0.3194) [16]. All samples were measured in triplicate under dark conditions. The color parameters were defined as follows: L*: lightness (0 = black, 100 = white); a*: green–red axis (−a* = greenness, +a* = redness); b*: blue–yellow axis (−b* = blueness, +b* = yellowness).

### 2.5. Determination of Oxidation

The conjugated diene value (CD) of oil samples was determined spectrophotometrically according to the standardized AOCS method (Ti 1a-64) with minor modifications [17]. The p-anisidine value (p-AV) was determined according to the standard method of the American Oil Chemists’ Society (AOCS Cd 18-90) [18]. The malondialdehyde (MDA) value, indicative of lipid peroxidation, was quantified using commercial kits (cat# BC0025, Solarbio) according to the previous method [15]. The acid value (AV), expressed as milligrams of potassium hydroxide (KOH) required to neutralize free fatty acids per gram of sample (mg KOH/g), was determined according to the American Oil Chemists’ Society (AOCS) method Cd 3d-63 with modifications [19]. The peroxide value (PV), expressed as milliequivalents of active oxygen per kilogram of sample (meq O_2_/kg), was determined according to the American Oil Chemists’ Society (AOCS) method Cd 8-53 with modifications [20]. The total oxidation value (TOTOX), reflecting the combined oxidative deterioration of lipids, was calculated using the formula:

(1) TOTOX = 2 × PV + AV

where PV is the peroxide value (meq active oxygen/kg sample) determined via AOCS Cd 8-53 (see Peroxide Value Determination) [20], and AV is the acid value (mg KOH/g sample) determined via AOCS Cd 3d-63 (see Acid Value Determination) [19].

### 2.6. Data Processing and Statistical Analysis

All data were analyzed using SPSS 26.0 (SPSS Inc., Chicago, IL, USA). Quantitative results are expressed as mean ± standard deviation (SD). Statistical significance was determined by one-way analysis of variance (ANOVA). Figures were generated using GraphPad Prism 10.0 (GraphPad Software Inc., San Diego, CA, USA), with distinct superscript letters (a, b, c) denoting significant differences (*p* < 0.05). The graphical abstract was created with Figdraw (https://www.figdraw.com).

## 3. Results

### 3.1. Degradation of Different Antioxidant Combinations

#### 3.1.1. DPPH Scavenging Efficiency

**Figure 1:**
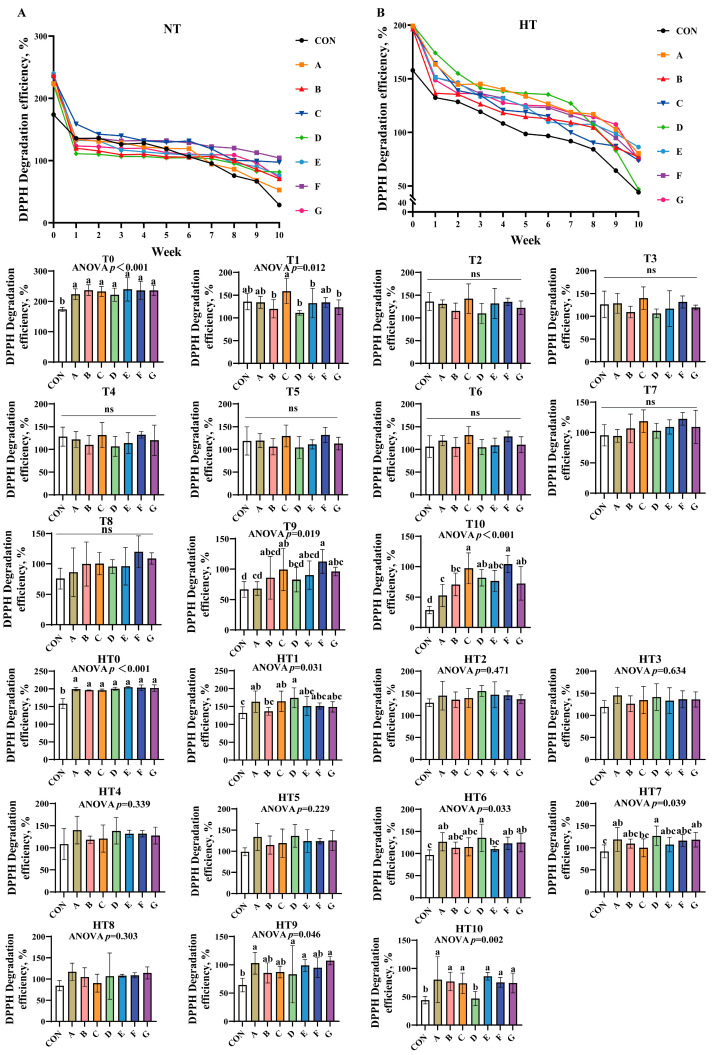
DPPH degradation efficiency in various combinations. DPPH degradation efficiency (%) in natural temperature ((**A**), NT), in high temperature ((**B**), HT). (T0–T10, corresponding to weeks 0–10 at normal temperature, HT0–HT10, corresponding to weeks 0–10 at high temperature). CON: Oxidized oil; A, 36 g/ton BHT; B, 60 g/ton EQ; C, 132 g/ton EQ; D, 10 g/ton EQ + 12 g/ton BHT; E, 10 g/ton EQ + 12 g/ton BHT + 6 g/ton CA; F, 20 g/ton EQ + 6 g/ton BHT + 6 g/ton CA; and G, 2 g/ton EQ + 25 g/ton BHT + 6 g/ton CA. Data with different letters in the same group are significantly different (*p* < 0.05), “ns” means not significant.

DPPH scavenging efficiency declined gradually across all treatments under both storage conditions (Figure 1A,B). At T0, all treatments significantly outperformed the control without intergroup differences. Group C showed the highest efficiency at T1, while C/A/E/F groups were comparable; B/D/G groups were significantly lower than C. No intergroup differences occurred from T2-T8. By T9, group F achieved peak efficiency (B/C/E/F/G comparable), while groups A and D declined markedly. At T10, groups C and F remained highest, followed by D/E/G; group B declined further and group A recorded the lowest values.

High-temperature conditions mirrored T0 trends at HT0. No significant intergroup differences were observed from HT1 to HT5. At HT6, group D displayed peak efficiency (A/B/C/F/G comparable), while group E declined. Group D maintained superiority over group C at HT7, with other groups matching control levels. HT8 showed no differences. A/D/G groups exhibited the highest efficiency at HT9, significantly surpassing control. Finally, A/B/C/E/F/G groups demonstrated comparable efficiency at HT10, significantly exceeding group D.

#### 3.1.2. ABTS Radical Absorbing Capacity

**Figure 2:**
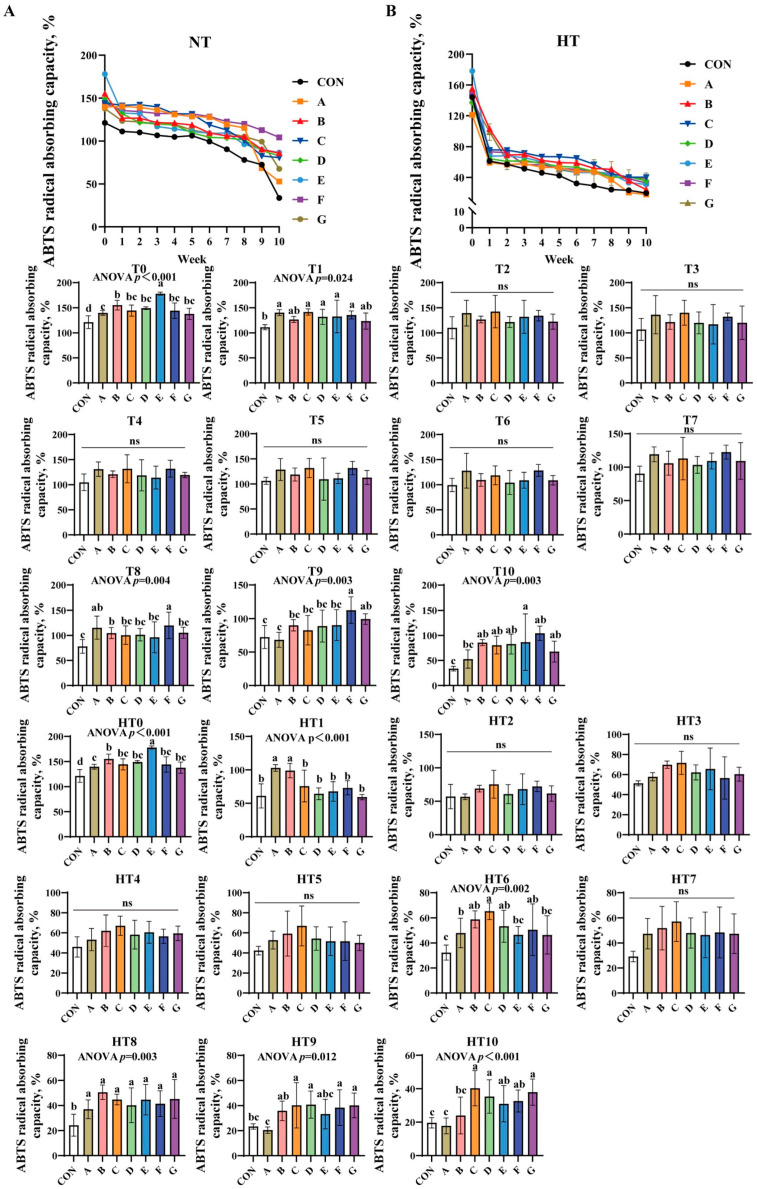
Decay of ABTS radical absorbing capacity in various combinations. ABTS radical absorbing capacity (%) in natural temperature ((**A**), NT), in high temperature ((**B**), HT). (T0–T10, corresponding to weeks 0–10 at normal temperature, HT0–HT10, corresponding to weeks 0–10 at high temperature). CON: Oxidized oil; A, 36 g/ton BHT; B, 60 g/ton EQ; C, 132 g/ton EQ; D, 10 g/ton EQ + 12 g/ton BHT; E, 10 g/ton EQ + 12 g/ton BHT + 6 g/ton CA; F, 20 g/ton EQ + 6 g/ton BHT + 6 g/ton CA; and G, 2 g/ton EQ + 25 g/ton BHT + 6 g/ton CA. Data with different letters in the same group are significantly different (*p* < 0.05), “ns” means not significant.

ABTS radical absorbing capacity progressively decreased across all treatments under both conditions (Figure 2A,B). At T0, groups E, B, and A showed sequentially lower values than control, while B/C/D/F/G and A/C/D/F/G subgroups exhibited comparable efficiency. Group E demonstrated the highest capacity at T1, with additive groups showing no statistical differences. No significant intergroup variations occurred from T2 to T7. By T8, groups A/F achieved the highest values, followed by group B, and then C/D/E/G (lowest), with AF and A/B/C/D/E/G subgroups showing comparable results. At T9, F/G groups showed highest efficiency, B/C/D/E groups intermediate, and group A lowest; F/G, B/C/D/E/G, and A/B/C/D/E subgroups remained statistically similar. By T10, group E exhibited peak efficiency, B/C/D/F/G groups showed comparable intermediate values, group A displayed marked reduction, and A/B/C/D/F/G subgroups were statistically similar with group A matching control levels.

High-temperature conditions replicated T0 trends at HT0. At HT1, A/B groups outperformed C/D/E/F/G groups, both surpassing control; A/B and C/D/E/F/G subgroups were comparable. No significant differences were observed from HT2 to HT5. At HT6, C/B/D/F groups showed highest efficiency versus control, followed by group A, with E/G groups lowest; C/B/D/F and A/B/D/E/F/G subgroups were comparable. HT7 revealed no intergroup differences. All treated groups significantly exceeded control at HT8 without intergroup variation. By HT9, C/D/F/G groups demonstrated peak efficiency versus control, B/C/D/E/F/G groups were comparable, and group A recorded lowest values. At HT10, C/D/G groups showed highest efficiency versus control, group B intermediate, group A lowest; C/D/E/F/G, B/E/F, and A/B subgroups exhibited no significant variations.

#### 3.1.3. Total Antioxidant Capacity (TAC)assay

**Figure 3:**
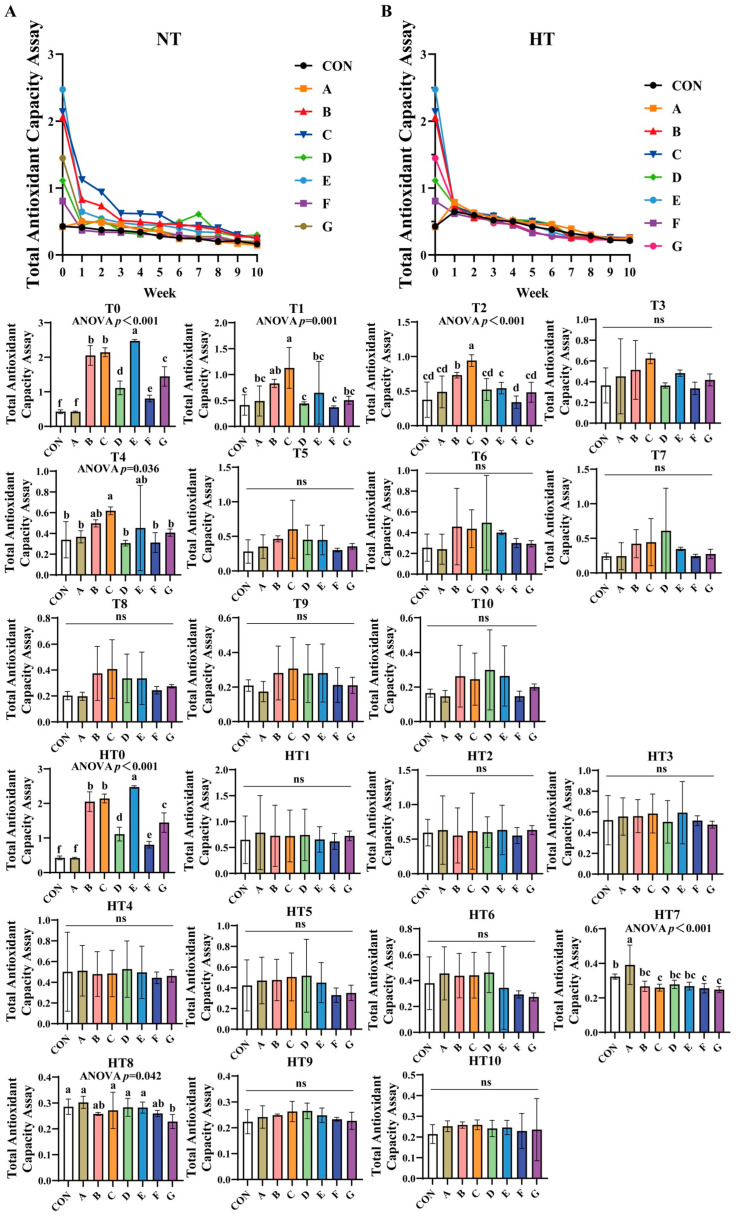
Decay of total antioxidant capacity assay in various combinations. Total Antioxidant Capacity Assay in natural temperature ((**A**), NT), in high temperature ((**B**), HT). (T0–T10, corresponding to weeks 0–10 at normal Temperature, HT0–HT10, corresponding to weeks 0–10 at high temperature). CON: Oxidized oil; A, 36 g/ton BHT; B, 60 g/ton EQ; C, 132 g/ton EQ; D, 10 g/ton EQ + 12 g/ton BHT; E, 10 g/ton EQ + 12 g/ton BHT + 6 g/ton CA; F, 20 g/ton EQ + 6 g/ton BHT + 6 g/ton CA; and G, 2 g/ton EQ + 25 g/ton BHT + 6 g/ton CA. Data with different letters in the same group are significantly different (*p* < 0.05), “ns” means not significant.

TAC progressively declined across all treatments under both storage conditions (Figure 3A,B). Under natural oxidation: At T0, groups E (highest), B/C, G, D, F, and A (lowest) showed sequential reduction versus control, with significant differences between B and C, and between A and control. By T1, C/B groups demonstrated superior efficiency, followed by A/E/G groups, and D/F groups exhibiting the lowest values; all subgroup pairs were comparable. At T2, efficiency declined sequentially from group C to B, E, and F, while A/D/E/F/G subgroups showed no differences. By T4, C/B/E groups outperformed A/D/F/G groups, with both subgroups internally comparable. No intergroup differences occurred from T3 to T10 except T4.

Under high-temperature stress: HT0 replicated T0 trends. No variations were observed during HT1-HT6. At HT7, group A achieved highest efficiency, B/D/E groups intermediate, and C/F/G groups lowest, with B/D/E/C/F/G subgroups comparable. By HT8, A/C/D/E groups showed superior efficiency, followed by B/C/F groups, and group G being least effective; A/B/C/D/E/F and B/F/G subgroups remained similar. No differences were detected from HT9 to HT10.

### 3.2. The Effects of Antioxidant Combinations on Color Variation

#### 3.2.1. The Effects of Antioxidant Combinations on L* Values

**Figure 4:**
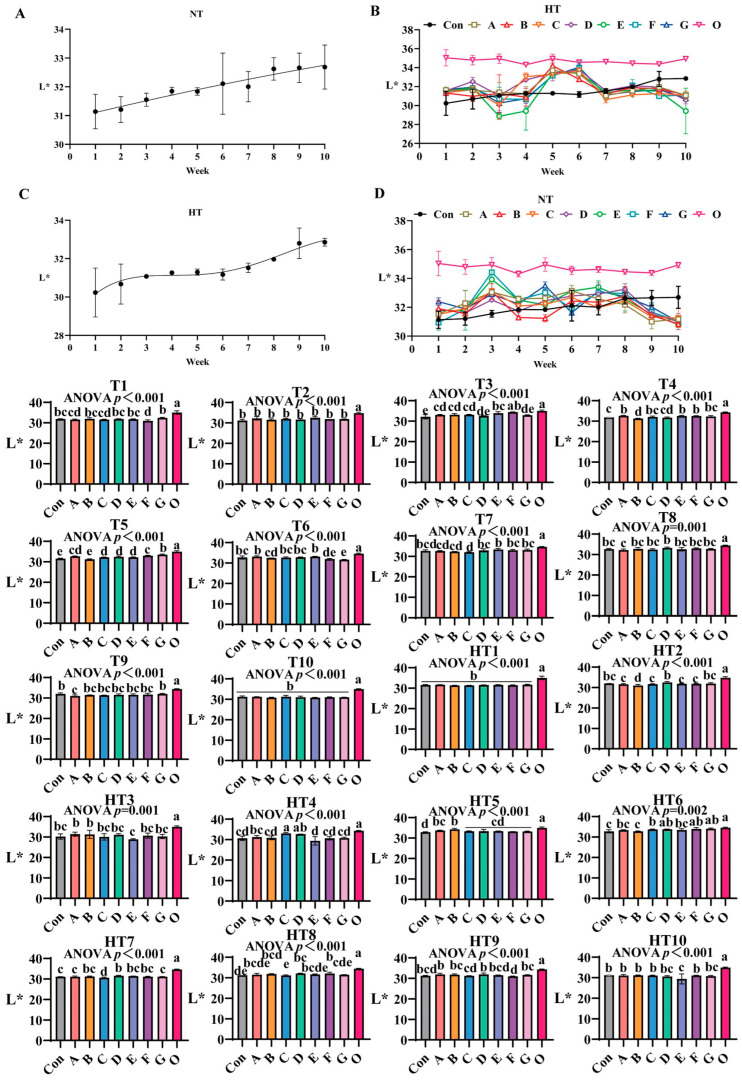
The influence of various antioxidant combinations on L*. (**A**) Oxidized oil without antioxidants under ambient storage conditions, NT; (**B**) antioxidant-fortified oils under ambient storage condition, NT; (**C**) oxidation oil without antioxidants under accelerated temperature, HT; (**D**) antioxidant-treated oils under accelerated oxidation, HT. T0–T10, corresponding to weeks 0–10 at normal Temperature, HT0–HT10, corresponding to weeks 0–10 at high temperature. Con: Oxidized oil; A, 36 g/ton BHT; B, 60 g/ton EQ; C, 132 g/ton EQ; D, 10 g/ton EQ + 12 g/ton BHT; E, 10 g/ton EQ + 12 g/ton BHT + 6 g/ton CA; F, 20 g/ton EQ + 6 g/ton BHT + 6 g/ton CA; and G, 2 g/ton EQ + 25 g/ton BHT + 6 g/ton CA; O (fresh oil) represents the pre-storage oxidation baseline. Data with different letters in the same group are significantly different (*p* < 0.05), “ns” means not significant.

L* values progressively increased during storage (Figure 4A–D), with fresh oil (O) maintaining the highest initial values. Under natural storage: At T1, group G exhibited significantly elevated values versus control, while A/B/C/D/E, B/D/E/G and A/C/F groups showed comparable results. No intergroup variations occurred at T2. By T3, group F demonstrated superior performance, significantly surpassing A/B/C and D/G groups (which were statistically similar), with E/F groups being equivalent. At T4, groups A, E and F emerged as top performers (statistically equivalent to A/C/E/F/G clusters), whereas group B showed the lowest values (similar to group D). During T5, values followed a sequential reduction: group G retained highest values, followed by group F, then statistically indistinguishable A/C/D/E groups, and finally group B; homogeneous A/C/D/E and A/F subgroups were identified. By T6, groups A/E maintained highest values, followed sequentially by B, F and G, with A/C/D/E, B/F and F/G clusters showing no internal differences. At T7, group E recorded maximal values, followed by comparable A/B groups, and group C showed minimal values; A/B/D/E/F/G and A/B/C groups were equivalent to control. Group D demonstrated optimal efficacy at T8 (B/C/E/F/G groups similar), while group A displayed lowest values (A/B/C/E/F/G equivalent). By T9, group G exhibited maximal preservation, group A minimal effectiveness, and intermediate groups B-F formed a homogeneous cluster (equivalent to control). All groups converged to equivalent values at T10.

Under high-temperature exposure: No intergroup differences occurred at HT1. HT2 identified group D as most effective, outperforming groups A–G, with A/C/E/F/G and D/G comparable to control. HT3 revealed comparable performance among A/B/C/D/F/G groups, while A/B surpassed group E. HT4 exhibited a value gradient: C/D groups (statistically equivalent) showed highest values, followed by group A, then comparable B/F/G groups, with group E being least effective; A/B/F/G and B/E/F/G clusters maintained internal consistency. At HT5, group B showed highest efficacy, C/D/E/F/G groups were comparable, and A/B, A/C/D/E/F/G clusters homogeneous. HT6 analysis ranked F/G groups as top performers, group B as least effective, and C/D/E/F/G along with A/B/E-control pairs as equivalent. HT7 confirmed group D’s dominance, followed by G/A groups, and group C showing minimal values; B/D/E/F and A/B/E/F/G subgroups were comparable. At HT8, group F ranked highest, group G intermediate, and group C lowest, with A/B/D/E/F and A/C/E/G clusters equivalent. No significant variations occurred at HT9 or HT10.

#### 3.2.2. The Effects of Different Antioxidant Combinations on a*

**Figure 5:**
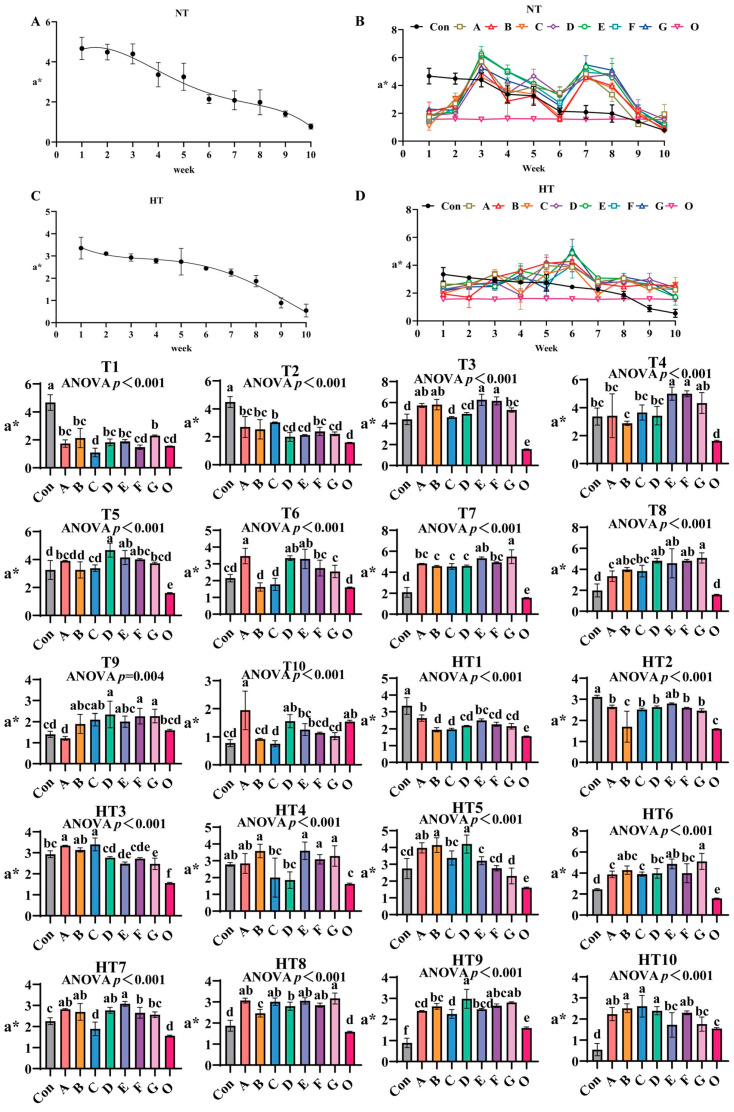
The effects of different antioxidant combinations on a* values. (**A**) Oxidized oil without antioxidants under ambient storage conditions, NT; (**B**) antioxidant-fortified oils under ambient storage condition, NT; (**C**) oxidation oil without antioxidants under accelerated temperature, HT; (**D**) antioxidant-treated oils under accelerated oxidation, HT. T0–T10, corresponding to weeks 0–10 at normal Temperature, HT0–HT10, corresponding to weeks 0–10 at high temperature. Con: Oxidized oil; A, 36 g/ton BHT; B, 60 g/ton EQ; C, 132 g/ton EQ; D, 10 g/ton EQ + 12 g/ton BHT; E, 10 g/ton EQ + 12 g/ton BHT + 6 g/ton CA; F, 20 g/ton EQ + 6 g/ton BHT + 6 g/ton CA; and G, 2 g/ton EQ + 25 g/ton BHT + 6 g/ton CA; O (fresh oil) represents the pre-storage oxidation baseline. Data with different letters in the same group are significantly different (*p* < 0.05), “ns” means not significant.

a* values progressively declined during ambient storage (Figure 5A–D), with fresh oil (Group O) as reference. Under natural storage: At T1, Group G showed highest values (surpassing A/B/D/E, *p* < 0.001), A/B/D/E/G groups comparable, Group F ranked second (similar to A/B/D/E/F), and C/F/O approached fresh oil levels. T2 featured Group C with maximal values (comparable to A/B/C/F, *p* > 0.05), significantly exceeding DEG (*p* < 0.001). At T3, E/F/A/B groups peaked without intragroup differences, followed by G/D groups, both surpassing Group C (*p* < 0.001). T4 showed EF groups highest (internally comparable), significantly exceeding A/B/C/D (*p* < 0.001). T5 had DEF groups maximal (comparable), followed by A/B/C/G, all surpassing Group O (*p* < 0.001). T6 demonstrated A/D/E groups peak values (comparable), exceeding F/G and B/C (*p* < 0.001). T7 revealed G/E groups as top performers, followed by A/F (E/G, A/E/F comparable), with B/C/D minimal (B/C/D/F comparable). T8 highlighted Group G highest (comparable to B/D/E/F/G), followed by A/C. T9 featured D/F/G maximal (comparable to B/C/D/E/F/G), surpassing Group A (*p* < 0.001), Group O matching control and ABCE. T10 showed Group A peak (comparable to A/D/O), E/F/G/B intermediate, Group C lowest (comparable to B/C/F/G).

Under high-temperature: HT1 had Group A highest (comparable to A/E), followed by D/F/G (B/C/D/F/G comparable), all surpassing Group O (*p* < 0.001). HT2: Group E highest, followed by A/C/D/F/G (comparable), Group B lowest (similar to O, *p* < 0.001). HT3: Groups A/C peak (comparable to B), followed by D (comparable to E/F), all exceeding O (*p* < 0.001). HT4: B/E/F/G highest (comparable), C/D reduced (comparable to A/control). HT5: Groups B/D maximal (comparable to A), followed by C, E significantly lower (*p* < 0.001). HT6: Group G highest (comparable to B/E), followed by D/F, A/C reduced. HT7: Group E peak (comparable to A/B/D), followed by F, C minimal (similar to O, *p* < 0.001). HT8: Group G highest (comparable to A/C/E/F), followed by D, both surpassing B/O (*p* < 0.001). HT9: Group D maximal (comparable to F/G), exceeding A/B/E, C reduced (comparable to A/E). HT10: Groups B/C/D highest (comparable to A/F), E/G reduced (comparable to A/F), all treatments maintained significantly higher values than Group O (*p* < 0.001), confirming antioxidant efficacy against thermal oxidation.

#### 3.2.3. The Impact of Different Antioxidant Combinations on b* Values

**Figure 6:**
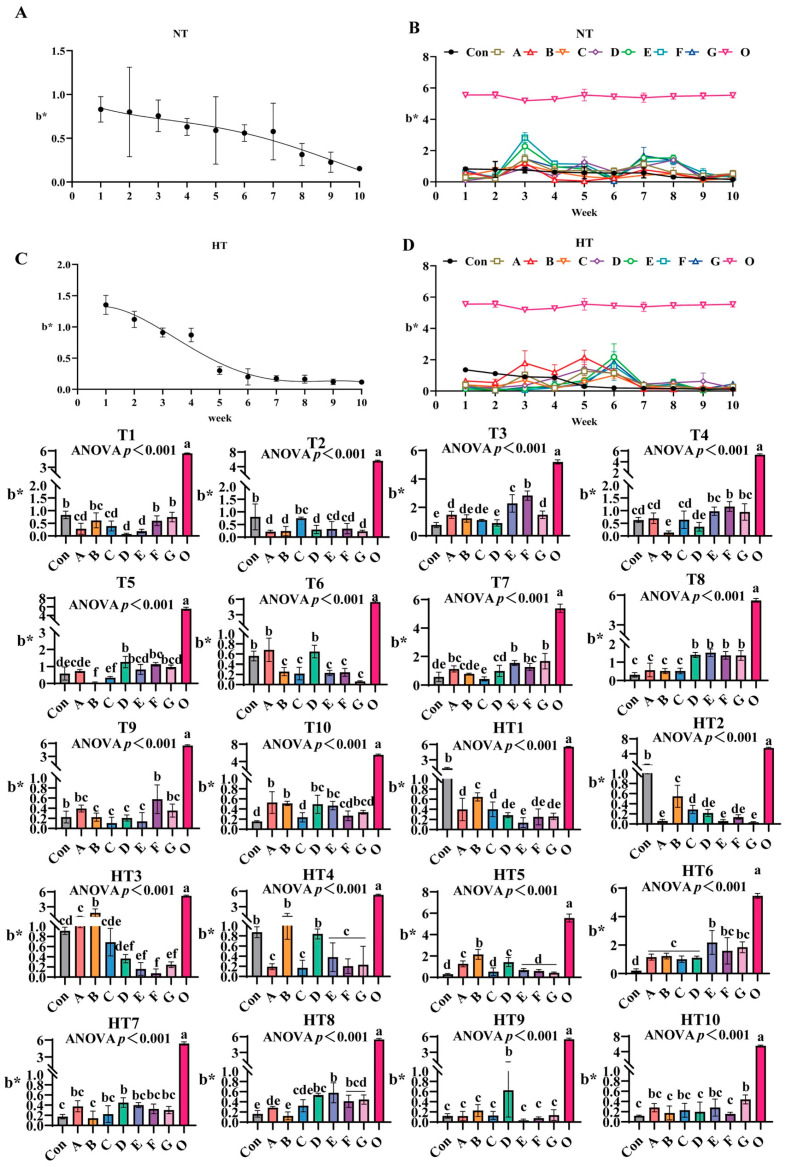
The changes in b* during lipid oxidation in feed and the effects of different antioxidant combinations on b*. (**A**) Oxidized oil without antioxidants under ambient storage conditions, NT; (**B**) antioxidant-fortified oils under ambient storage condition, NT; (**C**) oxidation oil without antioxidants under accelerated temperature, HT; (**D**) antioxidant-treated oils under accelerated oxidation, HT. T0–T10, corresponding to weeks 0–10 at normal temperature, HT0–HT10, corresponding to weeks 0–10 at high temperature. Con: Oxidized oil; A, 36 g/ton BHT; B, 60 g/ton EQ; C, 132 g/ton EQ; D, 10 g/ton EQ + 12 g/ton BHT; E, 10 g/ton EQ + 12 g/ton BHT + 6 g/ton CA; F, 20 g/ton EQ + 6 g/ton BHT + 6 g/ton CA; and G, 2 g/ton EQ + 25 g/ton BHT + 6 g/ton CA; O (fresh oil) represents the pre-storage oxidation baseline. Data with different letters in the same group are significantly different (*p* < 0.05), “ns” means not significant.

b* values progressively declined during ambient storage with fresh oil (Group O) as baseline (Figure 6A–D). Under natural storage: At T1, FG groups showed highest values (B/F/G comparable to control), A/B/C groups intermediate (intra-group comparable), D/E groups lowest (*p* < 0.001; D/E comparable). T2 featured Group C peak values (C/E/F comparable), A/B/D/G groups lowest (A/B/D/E/F/G comparable). At T3, Group F maximal, followed by E, then A/G groups, D minimal; A/B/C/G, B/C/D comparable. T4: Group F highest (E/F/G comparable), Group B poorest; A/C/E/G, A/C/D, B/D comparable. T5: Group D peak (D/E/F/G comparable), Group B minimal (B/C, A/B comparable). T6: A/D groups highest (intra-group comparable), significantly exceeding B/C/E/F/G (*p* < 0.001). T7: EG groups maximal, followed by A/E/F/G, then D groups (A/B/D/F, A/E/F/G comparable), Group C minimal (B/C comparable). T8: D/E/F/G highest (comparable), followed by A/B/C (consistent). T9: Group F peak (A/F/G comparable), B/C/D/E minimal (A/B/C/D/E/G comparable). T10: A/B highest (A/B/D/E/G comparable), significantly surpassing Group C (C/F/G comparable).

Under high-temperature: HT1: Group B highest, followed by A/C (A/C/D/F/G comparable), Group E lowest (D/E/F/G comparable). HT2: Group B peak, followed by C (C/D/F comparable), AEG minimal (*p* < 0.001; A/D/E/F/G comparable). HT3: Group B highest, followed by A (C comparable); Group F minimal (D/E/F/G comparable). HT4: B/D maximal (comparable), followed by A/C/E/F/G (consistent). HT5: Group B highest (A/D comparable), CEFG minimal (*p* < 0.001). HT6: Group E peak (E/F/G comparable), followed by A/B/C/D (A/B/C/D/F/G comparable). HT7: Group D maximal (A/C/D/E/F/G comparable), Group B minimal (A/B/C/E/F/G comparable). HT8: Group E highest (D/E/F/G comparable), followed by C, Group B lowest (A/B/C comparable). HT9: Group D peak, surpassing A/B/C/E/F/G (*p* < 0.001; internally consistent). HT10: Group G highest (A/C/E/G comparable), significantly exceeding B/D/F (*p* < 0.001; A/B/C/D/E/F comparable).

### 3.3. Impact of the Various Antioxidant Previously Combinations on the Variation in Conjugated Diene Value (CD)

**Figure 7:**
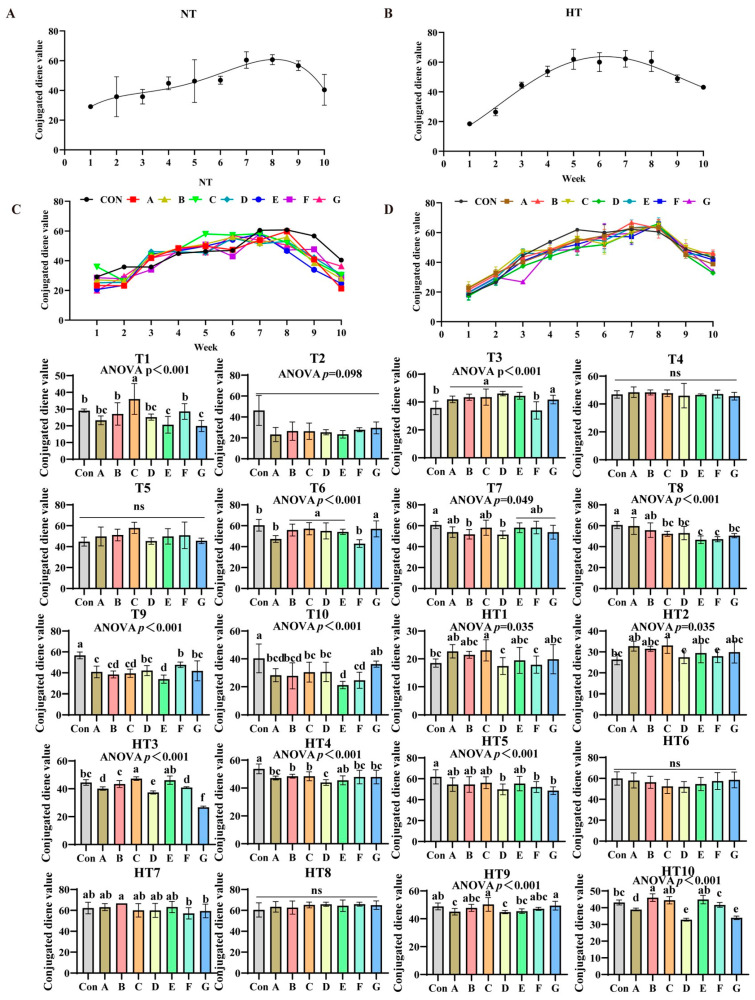
Impact of the various antioxidant formulations on the variation in conjugated diene value (CD). (**A**) Oxidized oil without antioxidants under ambient storage conditions, NT; (**B**) antioxidant-fortified oils under ambient storage condition, NT; (**C**) oxidation oil without antioxidants under accelerated temperature, HT; (**D**) antioxidant-treated oils under accelerated oxidation, HT. T0–T10, corresponding to weeks 0–10 at normal Temperature, HT0–HT10, corresponding to weeks 0–10 at high temperature. Con: Oxidized oil; A, 36 g/ton BHT; B, 60 g/ton EQ; C, 132 g/ton EQ; D, 10 g/ton EQ + 12 g/ton BHT; E, 10 g/ton EQ + 12 g/ton BHT + 6 g/ton CA; F, 20 g/ton EQ + 6 g/ton BHT + 6 g/ton CA; and G, 2 g/ton EQ + 25 g/ton BHT + 6 g/ton CA. Data with different letters in the same group are significantly different (*p* < 0.05), “ns” means not significant.

Conjugated diene values (CD) exhibited distinct oxidation patterns: ambient storage peaked at week 8 (Figure 7A,B), while high-temperature peaked earlier at week 5 (Figure 7C,D). At ambient storage: At T1, Group C showed highest values, followed by B/F groups, then A/D groups, with E/G lowest (*p* < 0.001); A/B/D/F and A/D/E/G groups comparable. T2 values slightly lower than control but non-significant. T3 revealed progressive decrease: A/B/C/D/E/G higher than F (*p* < 0.001), A/B/C/D/E/G comparable. No intergroup differences at T4–T5. T6: B/C/D/E/G higher than A/F (*p* < 0.001), A/B/C/D/E/G and A/F comparable. T7: A/C/E/F/G reduced versus control (*p* < 0.05), B/D further reduced; all groups comparable. T8: Group A highest (A/B comparable), followed by C/D/G, E/F lowest (*p* < 0.001); A/B, B/C/D/G, C/D/E/F/G comparable. T9: F/D/G highest, followed by A/B/C, E lowest (*p* < 0.001); D/F/G, A/B/C/D/G, B/C/E comparable. T10: G/C/D/A/B higher than F/E (*p* < 0.001); G/A/B/C/D, A/B/C/D/F, E/F comparable.

Under high-temperature storage: HT1-HT2: C/A/B/E/G higher than D/F (*p* < 0.001); C/A/B/E/G, B/D/E/F/G, D/F groups comparable. HT3: C/E highest, followed by B, then A/F, D/G lowest (*p* < 0.001); CE/AF comparable. HT4: E/F/G/A higher than B/C (*p* < 0.001), B higher than D; E/F/G/A and B/C comparable. HT5: A/B/C/E higher than D/F/G (non-significant). No differences at HT6/HT8. HT7: Group B higher than F/G (*p* < 0.05); A/B/C/D/E, A/C/D/E/F/G comparable. HT9: C/G highest, followed by E, then A/D (*p* < 0.001); C/G/B/F, A/B/D/E/F comparable. HT10: Group B highest, followed by F, then A/D/G (*p* < 0.001); B/C/E, D/G comparable.

### 3.4. The Process of Lipid Oxidation in Previously Basal Diet and the Effects of Different Combinations of Antioxidants on the Changes in p-Anisidine Value

**Figure 8:**
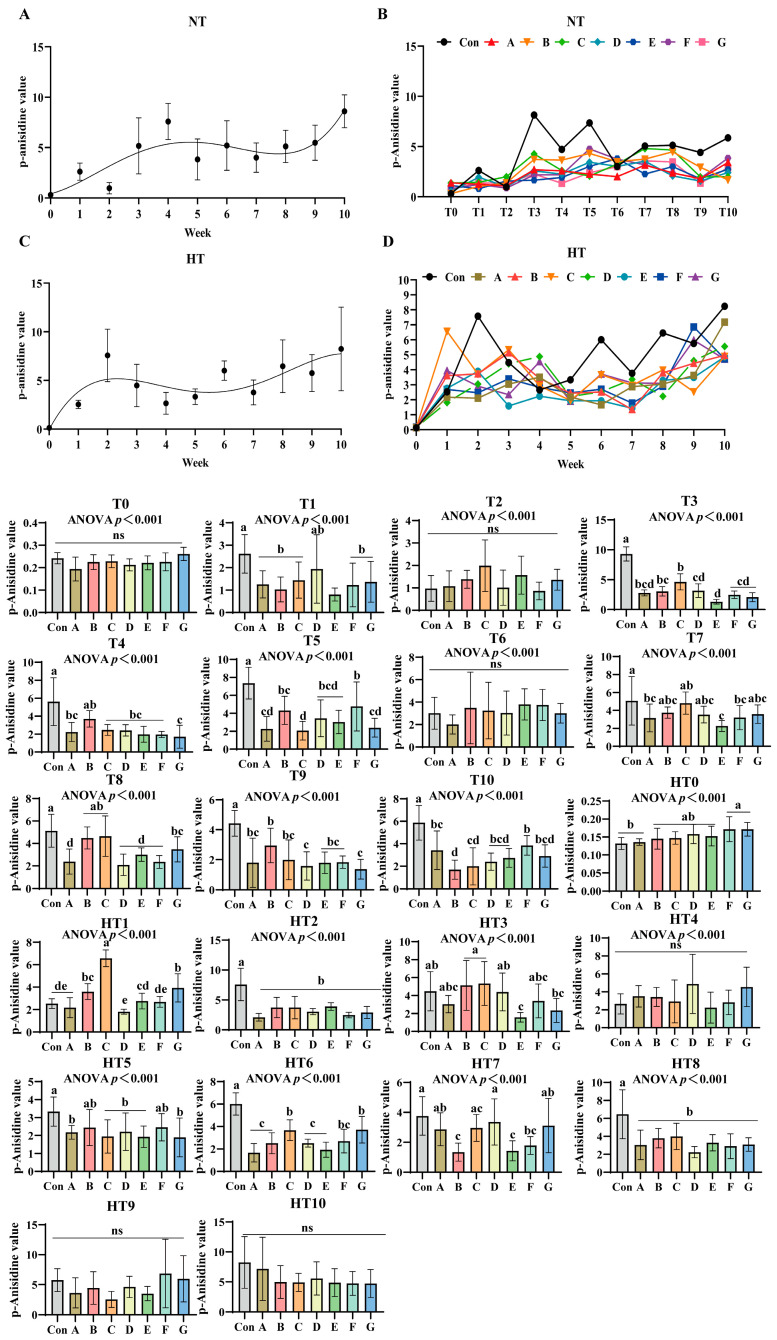
The process of lipid oxidation in animal feed and the effects of different combinations of antioxidants on the changes in p-anisidine value. (**A**) Oxidized oil without antioxidants under ambient storage conditions, NT; (**B**) antioxidant-fortified oils under ambient storage condition, NT; (**C**) oxidation oil without antioxidants under accelerated temperature, HT; (**D**) antioxidant-treated oils under accelerated oxidation, HT. T0–T10, corresponding to weeks 0–10 at normal Temperature, HT0–HT10, corresponding to weeks 0–10 at high temperature. Con: Oxidized oil; A, 36 g/ton BHT; B, 60 g/ton EQ; C, 132 g/ton EQ; D, 10 g/ton EQ + 12 g/ton BHT; E, 10 g/ton EQ + 12 g/ton BHT + 6 g/ton CA; F, 20 g/ton EQ + 6 g/ton BHT + 6 g/ton CA; and G, 2 g/ton EQ + 25 g/ton BHT + 6 g/ton CA. Data with different letters in the same group are significantly different (*p* < 0.05), “ns” means not significant.

p-Anisidine values (p-AV) increased progressively under both storage conditions (Figure 8A–D). No significant differences occurred at T0 or T2 compared to oxidized controls. Under ambient storage: T1 showed significant reductions in A/B/C/F/G and E groups (*p* < 0.001), with A/B/C/F/G exhibiting comparable values. At T3, values decreased sequentially from group C (highest) to B, then A/D/F/G, with E group lowest; A/B/C, A/B/D/F/G and A/D/E/F/G subgroups remained comparable. T4 featured Group B with maximal values, followed by statistically similar A/C/D/E/F groups, while Group G recorded minimal values. T5 demonstrated a descending trend: Group F (peak), followed by B/D/E groups, then A/G groups, with Group C lowest; F/B/D/E and A/G groups showed parity. No intergroup differences occurred at T6. T7 identified Group C with highest values and Group E with lowest, while A/B/C/D/F/G maintained comparability. T8 showed Group C superior, followed by C/B/G groups in decreasing order, while E/A/D/F groups exhibited further reduction with no intergroup variations. T9 featured Group B maximal, followed by A/C/E/G groups, with D/G groups showing additional decline; A/C/D/E/F/G subgroups were comparable. T10 recorded Group F with peak values, followed by A/G/E/D groups, and C/B groups with minimal readings; A/D/E/F/G and B/C/D/E/G showed no differences.

Under high-temperature: HT0 exhibited sequential reduction from F/G groups (highest) to B/C/D/E groups, then Group A; F/G and B/C/D/E subgroups were comparable. HT1 showed Group C maximal, followed by descending values in G, B, E, F, A, and D groups; B/G, B/E, E/F/A and A/D/F subgroups showed parity. HT2 values were significantly lower than control across all treatments without intergroup differences. HT3 displayed decreasing values from B/C groups to D group, then A/F groups, followed by G group, with E group lowest; A/B/C/D/F, A/D/F/G and A/E/F/G subgroups remained comparable. No differences occurred at HT4. HT5 featured B/F groups with highest values, followed by decreasing A/C/D/E/G groups; all treatments were comparable. HT6 showed descending values: B/G groups maximal, followed by F group, then B/D/A/E groups; B/G, C/F/G and A/B/D/E/F subgroups exhibited consistency. HT7 values decreased sequentially from Group D to G/A/C groups, then F group, with B/E groups lowest; A/C/D/G, A/C/F/G and B/E/F subgroups were comparable. HT8 treatments were significantly lower than control but intergroup comparable. No differences occurred at HT9 or HT10.

### 3.5. The Effects of Different Combinations of Antioxidants on Changes in Malondialdehyde Value (MDA)

**Figure 9:**
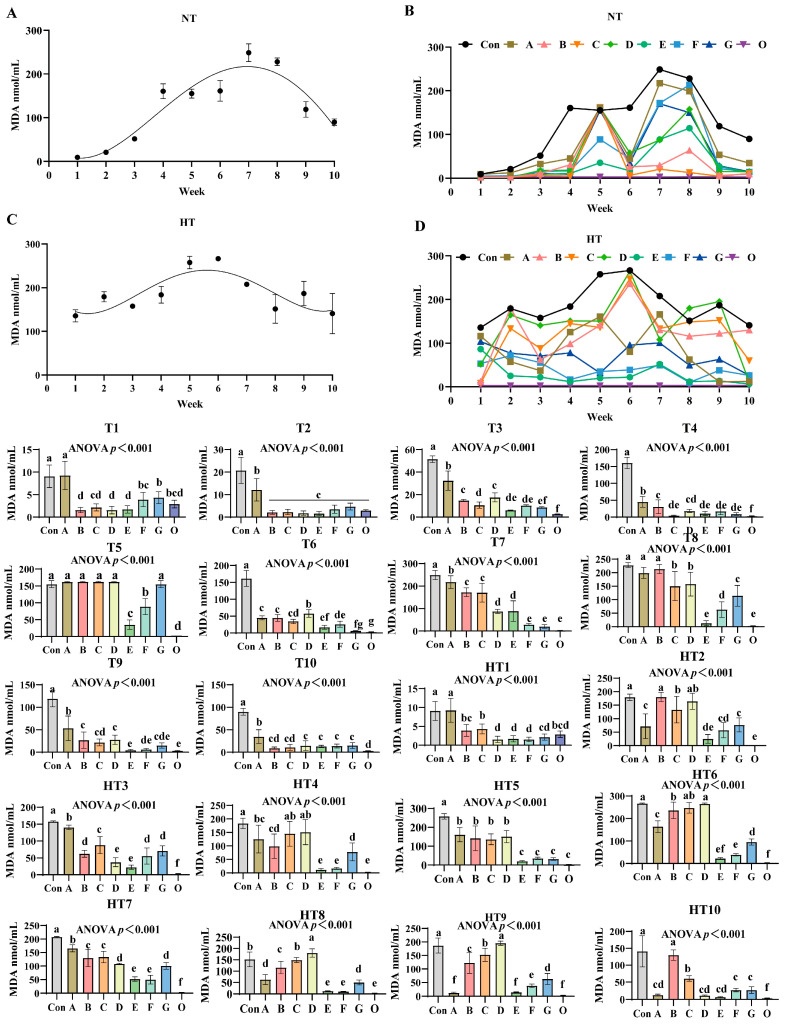
The effects of different combinations of antioxidants on changes in malondialdehyde (MDA) value. (**A**) Oxidized oil without antioxidants under ambient storage conditions, NT; (**B**) antioxidant-fortified oils under ambient storage condition, NT; (**C**) oxidation oil without antioxidants under accelerated temperature, HT; (**D**) antioxidant-treated oils under accelerated oxidation, HT. T0–T10, corresponding to weeks 0–10 at normal Temperature, HT0–HT10, corresponding to weeks 0–10 at high temperature. Con: Oxidized oil; A, 36 g/ton BHT; B, 60 g/ton EQ; C, 132 g/ton EQ; D, 10 g/ton EQ + 12 g/ton BHT; E, 10 g/ton EQ + 12 g/ton BHT + 6 g/ton CA; F, 20 g/ton EQ + 6 g/ton BHT + 6 g/ton CA; and G, 2 g/ton EQ + 25 g/ton BHT + 6 g/ton CA; O (fresh oil) represents the pre-storage oxidation baseline. Data with different letters in the same group are significantly different (*p* < 0.05), “ns” means not significant.

MDA values exhibited distinct oxidation kinetics: ambient storage peaked at week 7 (Figure 9A,B), high-temperature at week 6 (Figure 9C,D). Fresh oil (Group O) consistently demonstrated minimal oxidation. Under ambient storage: At T1, MDA reduction followed a sequential pattern: Group A showed highest values, followed by G, then F, C, and finally B/D/E groups versus control; F/G/O and B/C/D/E/O groups maintained comparable levels. T2 featured Group A with maximal values while other treatments showed significant reductions without intergroup differences. T3 revealed descending values: Group A highest, followed by D/B groups, then C, with F/G/E groups lowest; D/F/G, E/F/G and E/O groups were statistically similar. T4 values decreased progressively in Group A, B, D, F/E/G, O, though B/D and C/D/E/F/G groups showed parity. T5: A/B/C/D/G groups exhibited peak values, followed by F, then E, with Group O lowest; A/B/C/D groups were comparable. T6 levels declined sequentially from Group D to A/B groups, then C, F, E, G, and O; ABC, CF, EG and G/O groups showed no differences. T7: Progressive reduction from Group A to C/B groups, then E/D groups, finally F/G/O groups; B/C, D/E and F/G/O groups were comparable. T8: A/B groups maximal, followed by C/D groups, then G, F, and E/O groups; A/B and C/D groups-maintained consistency. T9 values reduced sequentially from Group A to D/B groups, then C/G/F groups, finally E/O groups; B/C/D/G, C/F/G and E/F/G/O groups were equivalent. T10: Group A highest, followed by D/E/F/G groups, with B/C/O groups lowest; B/C/D/E/F/G and B/C/O groups showed no variations.

Under high-temperature storage: HT1: Group A maximal, followed by C/B/O groups, then G, with D/E/F groups lowest; B/C/G/O and D/E/F/G/O groups were comparable. HT2 levels decreased sequentially from B/D groups to C group, then A/G/F groups, E group, and O group; B/D, C/D, A/F/G, E/F and E/O groups showed parity. HT3: Progressive reduction from Group A to C group, then B/G/F groups, followed by D/E groups, finally O group; B/F/G and D/E groups were similar. HT4: C/D groups peak, followed by A, then B, G, and E/F/O groups; A/C/D, A/B, B/G and E/F/O groups showed no differences. HT5 values declined from A/B/C/D groups to F/G/E/O groups; A/B/C/D and F/G/E/O groups exhibited comparable trends. HT6: Sequential decrease from D/C groups to B group, then A, G, F, E, and O group; C/D, B/C, E/F and E/O groups were equivalent. HT7: Group A maximal, followed by B/C groups, then G/D groups, E/F groups, and O group; B/C, D/G and E/F groups-maintained consistency. HT8 levels reduced progressively from D group to C group, then B group, A/G groups, finally E/F/O groups; E/F/O groups were comparable. HT9: Group D highest, followed by C, B, G, F, and A/E/O groups; A/E/O groups showed no variations. HT10: Sequential decline from B group to C group, then F/G groups, followed by A/D/E groups, with O group lowest; A/D/E/F/G groups exhibited comparable values.

### 3.6. The Effects of Different Antioxidant Combinations on Acid Value Changes

**Figure 10:**
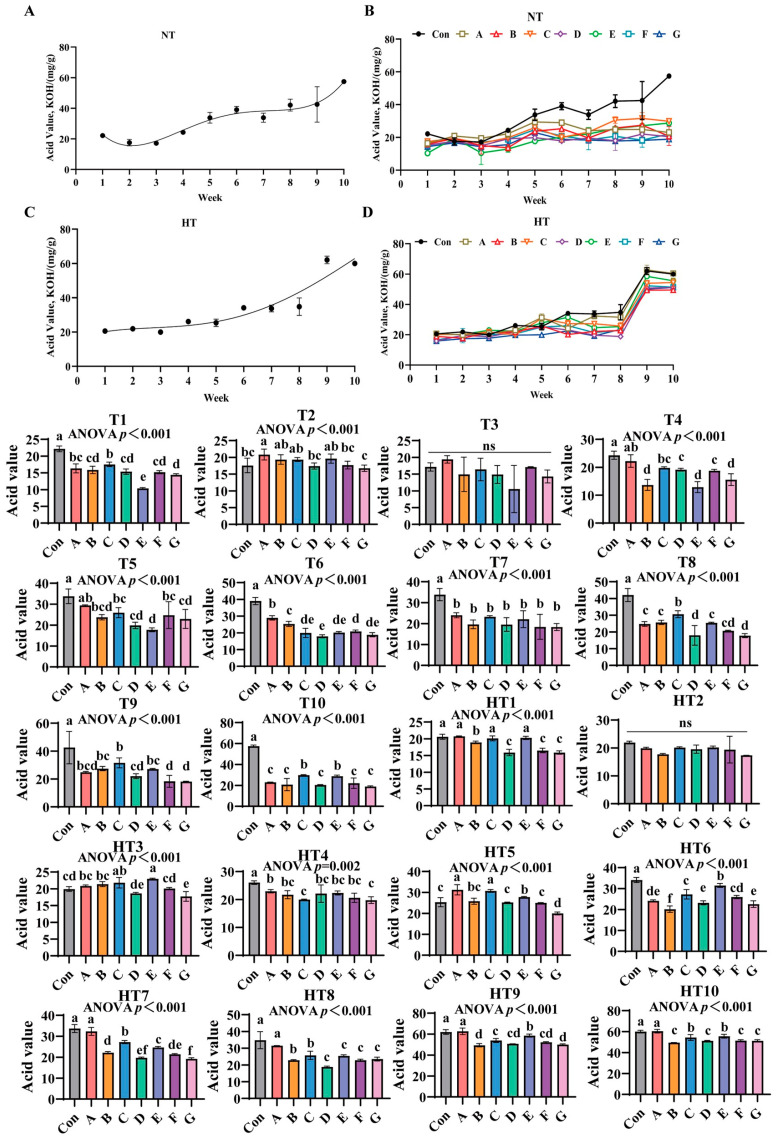
The effects of different antioxidant combinations on acid value changes. (**A**) Oxidized oil without antioxidants under ambient storage conditions, NT; (**B**) antioxidant-fortified oils under ambient storage condition, NT; (**C**) oxidation oil without antioxidants under accelerated temperature, HT; (**D**) antioxidant-treated oils under accelerated oxidation, HT. T0–T10, corresponding to weeks 0–10 at normal Temperature, HT0–HT10, corresponding to weeks 0–10 at high temperature. Con: Oxidized oil; A, 36 g/ton BHT; B, 60 g/ton EQ; C, 132 g/ton EQ; D, 10 g/ton EQ + 12 g/ton BHT; E, 10 g/ton EQ + 12 g/ton BHT + 6 g/ton CA; F, 20 g/ton EQ + 6 g/ton BHT + 6 g/ton CA; and G, 2 g/ton EQ + 25 g/ton BHT + 6 g/ton CA. Data with different letters in the same group are significantly different (*p* < 0.05), “ns” means not significant.

Acid value (AV) progressively increased in unprotected oils under both storage conditions (Figure 10A–D). Antioxidant efficacy varied significantly across treatments: under ambient storage: At T1, Group control showing highest values, followed by Group C, then B/D/F/G groups, and Group E demonstrating lowest values versus control; A/C and A/B/D/F/G groups maintained comparable levels. T2 exhibited Group A with maximal values, B/C/E groups intermediate, D/F groups lower, and Group G minimal; B/C/E and D/F/G groups were statistically similar. No intergroup differences occurred at T3. T4 values declined progressively: Group A highest, followed by C, then D/F groups, with G/B/E groups lowest; A/C, C/D/F and B/E/G groups showed parity. T5 reductions followed Group A (peak), then C/F/B groups, D/G groups, and Group E (minimal); A/C/F, B/C/D/F/G and B/D/E/G groups were comparable. T6 displayed descending values: Group A maximal, followed by B, then F, C/E/G groups, and Group D; C/E/F/G and C/D/E/G groups exhibited consistency. All treatments showed significantly lower AV than control at T7 without intergroup variation. T8 featured Group C with highest values, followed by A/B/E/F groups, then D/G groups; A/B/E/F and D/F/G groups were indistinguishable. T9 values ordered: Group C peak, followed by B/E/A groups, then D, and F/G groups; A/B/C/E and A/D/F/G groups lacked differences. T10 reductions occurred in C/E and A/B/D/F/G groups, with C/E and A/B/D/F/G groups comparable.

Under high-temperature storage: HT1 demonstrated sequential decline: A/C/E groups highest, followed by Group B, then D/F/G groups; A/C/E and D/F/G groups were statistically equivalent. HT2 showed no significant differences. HT3 values followed E/C groups maximal, then A/B/F groups, with D/E groups lowest; C/E, A/B/C, A/B/F, D/F and D/G groups-maintained similarity. HT4 revealed gradual decrease: A/B/D/E/F groups highest, followed by C/G groups; A/B/D/E/F and B/C/D/E/F/G groups were comparable. HT5 reductions featured A/B groups peak, followed by Group B, then D/F groups, and Group G; A/C, B/E and B/D/F groups showed no differences. HT6 displayed Group E with highest values, followed by C/F groups, then Group A, E/G groups, and Group B minimal; C/F, A/F and A/D/G groups were indistinguishable. HT7 ordered: Group A maximal, followed by C/E groups, then B, F, D, and Group G; B/F, D/F and D/G groups were comparable. HT8 reductions showed Group A highest, followed by B/C groups, then D/E/F/G groups; B/C and D/E/F/G groups exhibited parity. HT9 decline featured Group A peak, followed by E, then C/D/F groups, and B/G groups; C/D/F and B/D/F/G groups lacked significance. HT10 sequential decrease: Group A highest, followed by C/E groups, then B/D/F/G groups; C/E and B/D/F/G groups remained similar.

### 3.7. The Lipid Oxidation Process in Feed Oils and the Effects of Different Combinations of Antioxidants on Peroxide Value Changes

**Figure 11:**
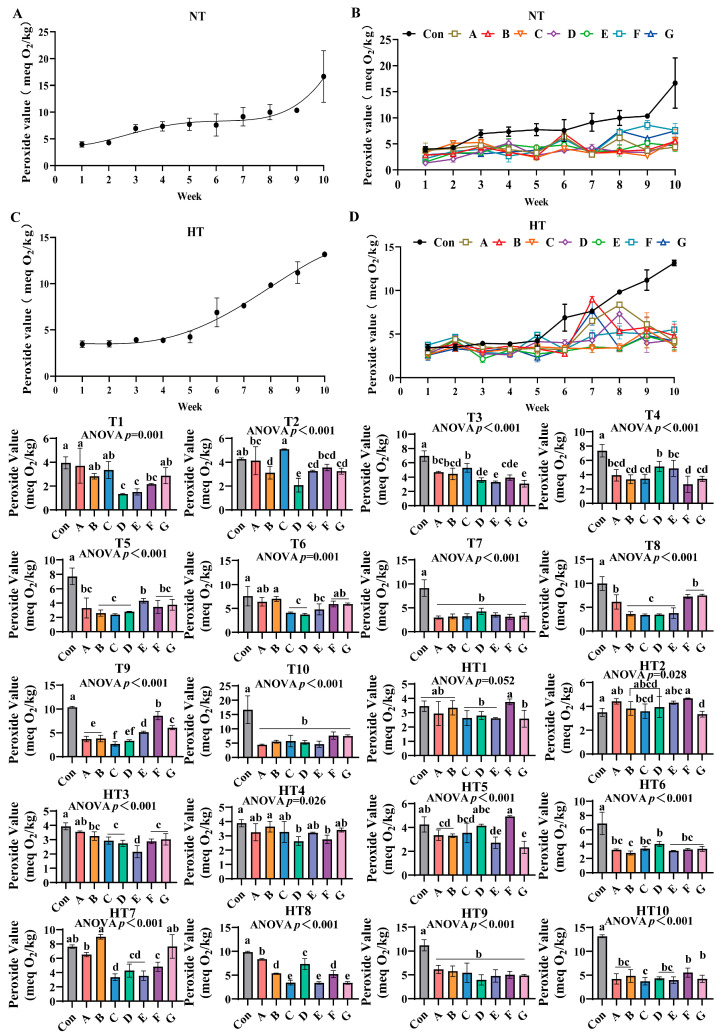
The effects of different combinations of antioxidants on peroxide value changes. (**A**) Oxidized oil without antioxidants under ambient storage conditions, NT; (**B**) antioxidant-fortified oils under ambient storage condition, NT; (**C**) oxidation oil without antioxidants under accelerated temperature, HT; (**D**) antioxidant-treated oils under accelerated oxidation, HT. T0–T10, corresponding to weeks 0–10 at normal Temperature, HT0–HT10, corresponding to weeks 0–10 at high temperature. Con: Oxidized oil; A, 36 g/ton BHT; B, 60 g/ton EQ; C, 132 g/ton EQ; D, 10 g/ton EQ + 12 g/ton BHT; E, 10 g/ton EQ + 12 g/ton BHT + 6 g/ton CA; F, 20 g/ton EQ + 6 g/ton BHT + 6 g/ton CA; and G, 2 g/ton EQ + 25 g/ton BHT + 6 g/ton CA. Data with different letters in the same group are significantly different (*p* < 0.05), “ns” means not significant.

Peroxide values progressively increased during storage under both conditions (Figure 11A–D). Ambient storage: At T1, Group A exhibited highest values with comparable levels among A/B/C/G groups. Group F demonstrated intermediate values, while Group D showed lowest values comparable to D/E/F groups. T2 featured Group C with peak values, followed sequentially by Group A, F, E, G, B and finally Group D; A, E/F/G and B, E/F/G subgroups maintained statistical equivalence. T3 showed Group C retaining maximal values, trailed by A/B groups, then Group F, with E/G groups exhibiting minimal values; A/B/C and A/B/F clusters showed parity. T4 displayed progressively lower values in D/F groups followed by B/C/G/F groups with intra-cluster consistency. T5 identified Group E with highest values, followed by B/C/D groups, while A/F/G groups demonstrated lower but comparable values. T6 featured Group B with peak values, followed by A/B/F/G groups, then Group E, C and finally D; A/B/F/G and C/D/E clusters showed overlapping values. T7 and T10 revealed all treatments significantly lower than controls without intergroup variations. T8 showed A/F/G groups superior to E/B/C/D groups with sequential reductions. T9 demonstrated Group F maximal, followed by G, E, A/B groups, and Group C, with Group D intermediate between A/B and C groups.

Under high-temperature storage: HT1: F/A/B groups exceeded C/D/E/G groups with intra-cluster comparability. HT2: F/A/E/B/D groups surpassed C/G groups. HT3: A/B groups maximal, followed by C/G/F groups, then Group D, with Group E minimal. HT4: Group B peak, trailed by A, C, E and G groups; D/F groups showed minimal values. HT5: Group F highest, followed by D, C, A, B, E and finally G with overlapping subgroups. HT6: D/C/A/G/F groups exceeded Group B. HT7: Group B maximal, followed by A, F and C. HT8: A/D groups surpassed B/F and C/E/G groups, respectively. HT9 mirrored ambient T7/T10 trends with treatments outperforming controls. HT10: F/G groups highest, followed by A, B, D and E groups; A/B/D/E/F/G and F/G/A/B/D/E clusters-maintained comparability. All experimental groups consistently demonstrated lower peroxide values than untreated controls throughout storage.

### 3.8. The Effects of Different Combinations of Antioxidants on Total Oxidation Value Changes

**Figure 12:**
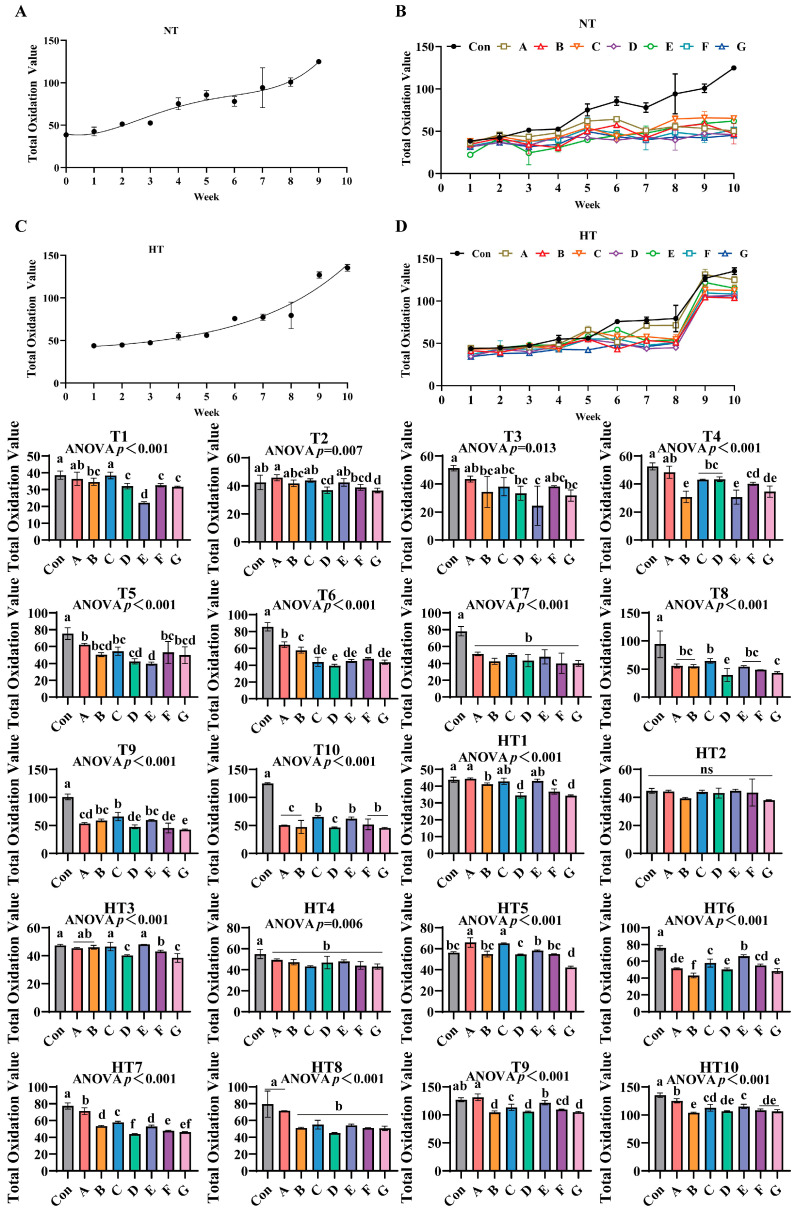
The effects of different combinations of antioxidants on total oxidation value changes. (**A**) Oxidized oil without antioxidants under ambient storage conditions, NT; (**B**) antioxidant-fortified oils under ambient storage condition, NT; (**C**) oxidation oil without antioxidants under accelerated temperature, HT; (**D**) antioxidant-treated oils under accelerated oxidation, HT. T0–T10, corresponding to weeks 0–10 at normal Temperature, HT0–HT10, corresponding to weeks 0–10 at high temperature. Con: Oxidized oil; A, 36 g/ton BHT; B, 60 g/ton EQ; C, 132 g/ton EQ; D, 10 g/ton EQ + 12 g/ton BHT; E, 10 g/ton EQ + 12 g/ton BHT + 6 g/ton CA; F, 20 g/ton EQ + 6 g/ton BHT + 6 g/ton CA; and G, 2 g/ton EQ + 25 g/ton BHT + 6 g/ton CA. Data with different letters in the same group are significantly different (*p* < 0.05), “ns” means not significant.

The total oxidation values of untreated control groups exhibited an increasing trend during both ambient and high-temperature storage (Figure 12A–D). In antioxidant-supplemented treatments, under T1, group C showed the highest total oxidation value, with no significant differences observed between groups A and C, followed by group B. Groups B, D, F, and G displayed comparable values, while groups D, F, and G were significantly lower than A/C, and group E exhibited the lowest value. For T2, group A recorded the highest oxidation value, with no significant differences among groups A, B, C, and E. Group D was significantly lower than A, while groups B, D, and F showed comparable levels, and group G displayed the lowest value with no differences among D, F, and G. In T3, group A maintained the highest oxidation value, followed by B, D, and G, though no significant differences were observed among groups A, B, C, D, F, and G, while group E remained the lowest. Under T4, group A exhibited the highest oxidation value, followed by C and D (no intergroup differences), while group F was lower than C/D and group G lower than F, with F/G showing comparable levels. Groups B and E displayed the lowest values, with no differences among B, E, and G. For T5, group A showed the highest oxidation value, with no differences among A, B, C, F, and G, followed by group D, while group E was the lowest, and B, D, E, and G exhibited comparable levels. In T6, group A recorded the highest value, followed by B and F, while group D was the lowest, with D/E/G and E/F/G showing no intergroup differences. T7 and T4 treatments demonstrated significantly lower oxidation values in all supplemented groups compared to controls, with no differences among treatments. For T8, group C displayed the highest oxidation value, followed by G (no differences among A, B, C, E, and F), while group D was the lowest. In T9, group C showed the highest value, followed by B and E (no differences among B, C, and E), while group A was lower (no differences among A, B, and E), followed by D and F (no differences among A, D, and F), with group G exhibiting the lowest value (no differences among D, F, and G). T10 revealed the highest oxidation values in groups C, E, F, and G (no intergroup differences), while A, B, and D showed significantly lower values with no differences among them.

Under high-temperature treatments, HT1 showed group A with the highest oxidation value (no differences among A, C, and E), followed by B and F, while D and G displayed the lowest levels (no intergroup differences). HT2 exhibited no significant differences among all groups. For HT3, groups C and E showed the highest values (no differences among A, B, C, and E), followed by F, while D and G were the lowest. HT4 demonstrated significantly reduced oxidation values in all supplemented groups compared to controls, with no intergroup differences. In HT5, groups A and C displayed the highest values, followed by E, D, and G (no differences among B, E, F and B, D, F), while group B was the lowest. For HT6, group E recorded the highest oxidation value, followed by C (no differences between C and F), then A (no differences between A and F), and D/G (no differences among A, D, and G), with group B as the lowest. HT7 showed group A with the highest value, followed sequentially by C, B/E, F, and D (no differences between D/G or F/G). HT8 exhibited group A as the highest, followed by B, C, D, E, F, and G (no intergroup differences). In HT9, group A displayed the highest value, followed by E, C, A/D/G (no differences between C/F or B/D/F/G), with group B as the lowest. HT10 revealed group A with the highest oxidation value, followed by E (no differences between C and E), then C (no differences among C, D, F, and G), and group B as the lowest (no differences among B, D, F, and G). All antioxidant-supplemented groups consistently exhibited lower oxidation values than untreated controls throughout storage.

## 4. Discussion

The oxidative stability of feed lipids, particularly those rich in polyunsaturated fatty acids (PUFAs), is a critical concern in animal nutrition due to their susceptibility to oxidative degradation. Numerous studies have demonstrated that the consumption of oxidized lipids can negatively affect meat and fish quality by promoting lipid peroxidation in muscle tissues, leading to increased thiobarbituric acid reactive substances levels, rancid odors, discoloration, and reduced shelf-life [21,22,23]. Additionally, oxidized feed can impair the deposition of essential fatty acids and antioxidants in tissues, thereby compromising the nutritional value and oxidative stability of the final products [24,25]. These carry-over effects highlight the importance of preventing lipid oxidation in feed to ensure both animal health and product quality.

Synthetic antioxidants such as BHT and ethoxyquin are widely used in feed and food systems and are regulated by authorities such as the European Food Safety Authority (EFSA) and the U.S. Food and Drug Administration (FDA), with clearly defined maximum allowable levels (e.g., BHT ≤ 200 g/ton in feed per Regulation (EC) No. 1831/2003). While these compounds are effective and cost-efficient, increasing consumer demand for clean-label and “natural” products has driven interest in plant-derived antioxidants, such as tocopherols and polyphenols. However, natural antioxidants may have limitations in oxidative stability, solubility, or consistency. This study systematically evaluated the efficacy of single and composite antioxidants under ambient and high-temperature storage conditions, focusing on their molecular structures, degradation kinetics, and synergistic interactions. The findings highlight the importance of antioxidant combinations in mitigating lipid oxidation and preserving feed quality.

The observed synergistic effects stem from the complementary mechanisms of the individual antioxidants: butylated hydroxytoluene (BHT) acts as a radical scavenger by donating hydrogen atoms to terminate lipid peroxidation chain reactions [26]; ethoxyquin (EQ) stabilizes free radicals through resonance delocalization within its aromatic structure, effectively inhibiting the propagation phase of oxidation [27]; and citric acid (CA) functions primarily via chelation, utilizing its carboxyl groups to sequester pro-oxidative metal ions (e.g., Fe^2+^, Cu^2+^) [28], thereby preventing metal-catalyzed lipid peroxidation. To comprehensively evaluate the oxidative stability of feed lipids under thermal stress, this study employed a multi-parameter system encompassing visual (L, a, b* color values) and chemical indicators of primary (conjugated dienes (CD), peroxide value (PV)), secondary (p-anisidine value (p-AV), malondialdehyde (MDA)), and total oxidation (total oxidation value (TOTOX = 2PV + p-AV), acid value (AV)). The results unequivocally demonstrate a fundamental temperature- and stage-dependence of antioxidant efficacy.

Lipid oxidation-induced discoloration manifests as decreased L (darkening) and increased a/b* (redness/yellowness) due to pigment degradation and polymer formation [29,30]. Accelerated storage caused significant darkening in controls, whereas the composite antioxidant Group F maintained higher L* and stable a*/b*, demonstrating effective visual preservation. Crucially, single antioxidants exhibited fundamental limitations: EQ/BHT-dominated formulations (e.g., Group C and B) preserved early-stage L* at ambient T1 through aromatic resonance delocalization quenching primary radicals but failed in late-stage metal control (evidenced by Group B’s poor HT9 performance). Conversely, pure citric acid systems lacked early radical scavenging capacity (Group F’s weak T1 performance) despite strong mid-late b* retention via Fe^2+^/Cu^2+^ sequestration suppressing carbonyls. High-temperature storage reshaped efficacy hierarchies: thermally accelerated hydroperoxide decomposition amplified citric acid’s role (Group F and E achieving peak a* at HT4 via metal chelation), while BHT’s volatility compromised performance (Group A’s severe HT8 L* loss). Rationally designed composites overcame these constraints: the ternary system Group E delivered synergistic temperature-stage adaptation and domination of BHT. EQ’s resonance delocalization terminated primary radicals (achieving dominant HT2 a*), BHT provided thermal resistance, and citric acid’s Fe^3+^/Cu^2+^ chelation suppressed alkoxyl radicals, yielding superior HT6 b* preservation. This synergy bridged critical gaps: citric acid extended EQ/BHT efficacy during ambient storage (Group E’s sustained T5-T6 L* stability) and prevented BHT volatilization under thermal stress. Dose optimization was pivotal—excessive EQ in Group C induced pro-oxidative b* loss at HT9, whereas Group E’s balanced formulation enhanced cross-stage performance at reduced total concentration. Thus, Group E emerges as the optimal solution, synchronizing radical scavenging for early L* protection and metal chelation for mid-late a*/b* stability across temperatures, effectively overcoming kinetic mismatches and dose-dependent risks.

Conjugated diene (CD) evolution demonstrates distinct stage- and temperature-dependent antioxidant efficacy [31]. Under ambient storage, synthetic radical scavengers (e.g., Group C) dominated early-stage protection (T1–T3), effectively stabilizing lipid radicals through aromatic resonance delocalization, with peak efficacy at T1. Conversely, metal chelators (e.g., Group F) excelled later at T3 by sequestering pro-oxidative metals. High-temperature storage accelerated oxidation kinetics, shifting peak CD occurrence to week 5 versus week 8 under ambient conditions. This acceleration reshaped antioxidant hierarchies, favoring chelators during critical mid-stages—evidenced at HT4 where Groups E, F, G, and A outperformed Groups B and C. Crucially, EQ/BHT+CA composites (represented by ADEG systems) exhibited synergistic resilience by balancing early radical quenching at ambient T1 (demonstrated by Groups A, B, D, F) with mid-stage metal control conferring stability at HT4. Direct comparisons confirm composite superiority: single EQ/BHT systems (Groups A, B) displayed early efficacy but suffered pro-oxidative CD rebound at HT9 and metal-catalysis vulnerability at HT4, while pure CA (Group F) showed weak early protection (highest T1 CD) despite late-stage strength. In contrast, EQ/BHT+CA composites (Groups ADEG, G) synchronized protective mechanisms—EQ/BHT inhibited primary oxidation while CA suppressed secondary oxidation and mitigated thermal pro-oxidation, enabling Group G to achieve lower CD than Group A at HT9. This synergy delivered stage-adaptive defense, providing superior early CD reduction versus CA alone and extending the induction period by 2 weeks relative to EQ/BHT systems under thermal stress. Thus, optimized ADEG-type composites emerge as the optimal solution, overcoming single-antioxidant limitations through synergistic, temperature-resilient protection.

The p-anisidine value (p-AV) specifically quantifies unsaturated aldehydes (e.g., 2-alkenals, 2,4-dienals) formed during late-stage lipid oxidation [18], providing critical insight into secondary oxidation dynamics. Results demonstrate antioxidant efficacy is fundamentally dictated by storage temperature and oxidation stage. Under ambient conditions, synthetic radical scavengers (EQ, BHT) dominated early protection, with Group C achieving the lowest p-AV at T3 via aromatic resonance delocalization quenching alkoxyl radicals. Conversely, chelator-enhanced composites (e.g., Group F) excelled in mid-late stages, significantly reducing p-AV versus controls at T5 through Fe^2+^/Cu^2+^ sequestration blocking hydroperoxide-derived carbonyl formation—consistent with MDA data and confirming superior secondary oxidation suppression after 60 days. High-temperature storage reshaped these dynamics: thermally accelerated aldehyde formation amplified pro-oxidative risks in single antioxidants, exemplified by Group B exhibiting the highest p-AV at HT9 due to compromised radical recycling. In contrast, CA-composites showed enhanced resilience: Group F maintained the lowest p-AV at HT3 versus Groups B and C by chelating metal catalysts of malondialdehyde precursors, while the ternary system Group E delivered cross-stage efficacy—dominating HT6 p-AV reduction through synergistic integration of EQ’s primary oxidation control, BHT’s peroxyl radical interception, and CA’s aldehyde suppression. Critical limitations of single antioxidants were evident: EQ/BHT groups failed late-stage aldehyde control (Group B: highest T9 p-AV from inadequate metal sequestration), while pure CA lacked early radical termination (permitting hydroperoxide accumulation). Composites bridged this gap: Group E significantly reduced p-AV versus Group B at HT9 via synchronized mechanisms—CA extended EQ/BHT’s early protection (demonstrated by ambient T5 efficacy) while EQ/BHT enhanced CA’s thermal stability (reflected in HT3 performance). Thus, Group E represents the optimal formulation, delivering stage-adaptive and temperature-resilient aldehyde control through complementary radical scavenging and metal deactivation, overcoming intrinsic limitations of single-component systems.

Malondialdehyde dynamics reflect terminal ω-6 PUFA peroxidation [32], exhibiting concentration peaks during active hydroperoxide decomposition and declines in advanced stages via aldol condensation, degradation, or volatilization. Composite Group F demonstrated slowest accumulation, confirming effective propagation suppression. Malondialdehyde progression revealed critical temperature-stage dependencies: synthetic radical scavengers dominated ambient early-stage control with Group C achieving minimal T3 values through aromatic resonance quenching of peroxyl radicals, while chelator-enhanced Group F reduced T6 values via Fe^2+^/Cu^2+^ sequestration blocking hydroperoxide conversion. High-temperature storage reconfigured hierarchies as thermal acceleration amplified single-antioxidant pro-oxidative risks—Group A exhibited HT1 and HT7 peaks from BHT volatility limitations. Conversely, ternary Group E maintained lowest HT6 values through synergistic EQ-mediated primary termination, BHT peroxyl interception, and CA suppression of metal-catalyzed β-scission. Direct comparisons exposed inherent limitations: EQ/BHT groups failed late-stage control with Group A showing highest T8 values, while pure CA systems permitted early accumulation as evidenced by Group F elevated T1 values. Group E bridged performance gaps, reducing HT9 values versus Group A with 3-week protection extension through three mechanisms: citric acid enhancement of EQ/BHT radical quenching demonstrated by lower T5 values than Group F, EQ/BHT compensation of CA thermal limitations evidenced by lower HT3 values than Group D, and ratio-optimized pro-oxidation prevention shown by decreased HT9 values compared to Group C increases. Thus, Group E delivers adaptive peroxidation control through synchronized early radical scavenging and late metal deactivation, overcoming single-component kinetic constraints.

Acid value quantifies hydrolytic and oxidative free fatty acids as a critical rancidity indicator [33]. Composite Group F demonstrated significantly lower values, confirming enhanced suppression of hydrolytic pathways and primary oxidation through synergistic mechanisms [34]. Efficacy exhibited distinct temperature-stage dependencies: synthetic radical scavengers dominated ambient early-stage protection with Group C achieving maximal suppression at T1-T4 via ethoxyquin’s aromatic resonance quenching of alkoxyl radicals. Chelator-composites prevailed in mid-late stages as Group E reduced values at T8 through citric acid’s dual Fe^2+^/Cu^2+^ sequestration and proton-donor catalysis. High-temperature storage reconfigured dynamics with thermal hydrolysis acceleration amplifying single-antioxidant deficiencies—Group A showed HT1/HT4 peaks due to butylated hydroxytoluene volatility and metal-chelation inability. Conversely, ternary Group E maintained minimal HT6 values through synergistic ethoxyquin radical stabilization, butylated hydroxytoluene peroxyl interception, and citric acid suppression of metal-activated lipases. Critical limitations emerged: ethoxyquin/butylated hydroxytoluene groups failed late-stage control with Group C showing highest T9-T10 values; pure citric acid permitted early acidogenesis with Group F exhibiting elevated T2 values. Group E bridged gaps through significant HT9 reduction versus Group A, achieving two-week hydrolytic stability extension under thermal stress at 30% lower dosage than Group C. Mechanistic drivers included: citric acid-enhanced radical quenching demonstrated by lower T5 values than Group F; ethoxyquin/butylated hydroxytoluene compensation of thermal limitations evidenced by lower HT3 values than Group D; and ratio-optimized prevention of pro-hydrolytic effects shown by decreased HT10 values versus Group C increases. Thus, Group E delivers stage-adaptive rancidity control via synchronized early radical scavenging and late metal/lipase deactivation, overcoming single-component deficiencies across thermal regimes.

Peroxide value quantification showed significantly slower hydroperoxide accumulation in composite Group F versus single antioxidants, confirming superior initial oxidation inhibition and synergistic protection at reduced concentrations [35,36]. Temperature-stage dependencies emerged: synthetic radical scavengers dominated ambient early protection with Group D achieving minimal T1 values through ethoxyquin/butylated hydroxytoluene resonance-stabilized peroxyl radical quenching. Chelator-enhanced composites reduced T5 values during T4–T9 via Fe^2+^/Cu^2+^ sequestration blocking metal-catalyzed formation. High-temperature storage reconfigured hierarchies—Group A exhibited HT1/HT4 peaks from volatility and metal-chelation inability. Ternary Group E maintained minimal HT6 values through integrated synergy: EQ aromatic delocalization stabilizing alkyl radicals; BHT phenolic groups intercepting peroxyl radicals; CA carboxylate coordination suppressing metal-catalyzed generation. Critical deficiencies: EQ/BHT groups failed late control with Group C showing highest T3/HT3 values; pure CA permitted early accumulation with Group F elevated HT2 values. Group E bridged gaps delivering lower HT9 values versus Group A, three-week protection extension, and reduction versus Group C through CA-enhanced radical quenching demonstrated by lower T4 values than Group F; EQ/BHT thermal stability compensation evidenced by lower HT3 values than Group D; ratio optimization preventing pro-oxidation shown by decreased HT7 values versus Group C increases. Thus, Group E delivers stage-adaptive control via synchronized radical termination for early suppression and metal deactivation for mid-late stability, overcoming single-component limitations.

TOTOX value integrates primary oxidation measured by peroxide value and secondary oxidation quantified by p-anisidine value to capture complete oxidative deterioration. Values below 10 are considered acceptable for cooking oils [14,37]. The composite formulation Group E containing ethoxyquin, butylated hydroxytoluene, and citric acid delivered superior full-spectrum protection through synergistic radical scavenging, metal chelation, and hydroperoxide stabilization [38,39]. Efficacy demonstrated distinct temperature-stage dependencies: under ambient storage, synthetic scavengers dominated early protection with Group D achieving minimal TOTOX values at T1 via resonance-stabilized peroxyl radical quenching. Citric acid-enhanced composites prevailed during mid-late stages with Group E significantly reducing T9 values through ferrous and cupric ion sequestration suppressing secondary oxidation. High-temperature storage reconfigured antioxidant performance hierarchies. Thermal acceleration amplified single-antioxidant deficiencies as evidenced by Group A exhibiting peak values at HT1 and HT4. Conversely, ternary composites exhibited cross-stage resilience: Group E maintained minimal HT6 values through integrated mechanisms where ethoxyquin terminates alkyl radicals, butylated hydroxytoluene intercepts peroxyl radicals, and citric acid suppresses metal-activated decomposition. This system bridged critical gaps in single-antioxidant performance: ethoxyquin/butylated hydroxytoluene formulations showed late-stage collapse with Group C displaying highest T3 and HT3 values, while pure citric acid systems demonstrated early vulnerability with Group F exhibiting elevated T2 values. Group E delivered lower values than Group A at HT9, extended stability duration by four weeks, and achieved reduction versus Group C through citric acid-enhanced radical quenching confirmed by lower T4 values than Group F; ethoxyquin/butylated hydroxytoluene thermal stabilization evidenced by lower HT3 values than Group D; and optimized antioxidant ratios preventing pro-oxidative effects. These results validate Group E’s practical superiority for feed applications at reduced effective dosages.

Optimizing feed lipid antioxidants requires integrating advanced analytics with synergistic formulations. Conventional parameters offer useful oxidation indicators but lack sensitivity and early-detection capability, necessitating complementary techniques like FTIR spectroscopy and hydroperoxide value determination. This integrated approach enables holistic stage-specific assessment, precise mechanism differentiation, and improved formulation design. Crucially, compound systems (BHT/EQ/citric acid) outperform single additives, suppressing key markers—acid value, peroxide value, malondialdehyde—at reduced dosages. Synergy lowers additive inputs and costs while ensuring regulatory compliance, delivering extended shelf-life, minimized loss of PUFAs/fat-soluble vitamins, and reduced toxic compound accumulation. Future priorities include: (1) Optimizing synergistic ratios via RSM and machine learning for specific lipid matrices [40]; (2) validating bioavailability and oxidative stress modulation in poultry [41]; (3) assessing robustness across variable conditions (temperature, humidity, light) and fat sources (tallow, soybean oil, poultry fat) [42,43,44]; (4) developing natural-synthetic hybrids (e.g., rosemary extract/tocopherols with low-dose synthetics) for clean-label efficacy. Demonstrated efficiency supports broad industry adoption [45]; future advances require precision formulations validated across diverse application scenarios.

Validated efficiency of compound antioxidants at reduced concentrations supports broad feed industry adoption. Future advancements require precision formulation, mechanistic validation, and robust evaluation frameworks across application scenarios.

## 5. Conclusions

This study establishes that the rationally formulated composite antioxidant (Group E) delivers superior, stage-adaptive protection against feed lipid oxidation through synergistic integration of radical scavenging and metal chelation. The ternary system comprehensively controls primary oxidation, suppresses secondary degradation pathways, and maintains sensory quality by simultaneously quenching initiating radicals, stabilizing hydroperoxides, and sequestering pro-oxidative metals. Crucially, it resolves temperature-dependent limitations of single antioxidants: citric acid extends early-stage efficacy under ambient conditions, while ethoxyquin/BHT compensate thermal vulnerability at elevated temperatures. With optimized ratios preventing pro-oxidative effects, this approach achieves significant dose reduction and shelf-life extension, providing an industrially viable paradigm for enhancing oxidative stability in perishable lipid matrices.

## Figures and Tables

**Table 1:** The purity and effective dosage levels in different antioxidants combinations.

Treatment	BHT (99%)			EQ (95%)			CA (99%)		
	Purity (%)	Effective Content (g/ton)	Addition Volume in Diet (g/ton)	Purity (%)	Effective Content (g/ton)	Addition Volume in Diet (g/ton)	Purity (%)	Effective Content (g/ton)	Addition Volume in Diet (g/ton)
A	18	36	36.36						
B				30	60	63.16			
C				66	132	138.95			
D	6	12		5	10	10.53			
E	6	12		5	10	10.53	3	6	6.06
F	3	6		10	20	21.05	3	6	6.06
G	12.5	25		1	2	2.11	3	6	6.06

## Data Availability

The original contributions presented in this study are included in the article and Supplementary Material. Further inquiries can be directed to the corresponding author.

## Supplementary Materials

The following supporting information can be downloaded at: https://www.mdpi.com/article/10.3390/antiox14080981/s1, Table S1: Composition and nutrient levels of the basal diet (% dry matter).
