# Supplementary material for: Synergistic Effects of Antioxidant Blends: A Comparative Study on Oxidative Stability of Lipids in Feed Matrices
Source: Antioxidants (Basel). 2025 Aug 10;14(8):981. doi: 10.3390/antiox14080981 (PMC12382763; doi:10.3390/antiox14080981)
Supplement: Supplementary file 1 [file antioxidants-14-00981-s001.zip › antioxidants-3775794-supplementary.pdf]

Supplementary Table S1. Composition and nutrient levels of the basal diet (% dry matter).

Ingredients % as fed basis	week 0~3	week 4~6
Corn grain	9.4	45.3
Wheat grain	20.0	20.0
Corn gluten meal	0	6.0
Broken rice	30.0	0
Soybean meal	31.0	13.4
Cottonseed meal	3.00	6.00
Feather meal	1.00	1.50
Soybean oil	1.20	3.30
Dicalcium phosphate	1.37	1.05
Limestone	1.30	1.40
Sodium chloride	0.20	0.20
Sodium bicarbonate	0.25	0.25
Vitamins premix[1]	0.03	0.03
Mineral premix[2]	0.20	0.20
Choline chloride (50%)	0.16	0.16
Phytase (10,000 U/g)	0.02	0.02
L-Lysine hydrochloride (78%)	0.38	0.58
DL- Methionine (98%)	0.28	0.17
L-Threonine (99%)	0.15	0.12
L-Arginine Hydrochloride (98%)	0.02	0.12
L-Valine (98%)	0.04	0
L-Isoleucine (98%)	0	0.10
Total	100	100
Nutrient Parameters[3]		
Metabolizable energy, Mcal/kg	2.93	3.15
Crude protein, %	22.06	20.1
Total Ca, %	0.94	0.86
Available P, %	0.37	0.30
Digestible Lys, %	1.20	1.15
Digestible Met, %	0.54	0.48
Digestible Try, %	0.22	0.19
Digestible Thr, %	0.79	0.79
Digestible Met + Cys, %	0.87	0.89
Digestible Val, %	0.90	0.90
Digestible Arg, %	1.29	1.22
Digestible Iso, %	0.82	0.80

Note: [1] The vitamin premix provided (per kilogram of diets) the following: vitamin A, 15,000 IU; vitamin D3, 3600 IU; vitamin E, 30 IU; vitamin K3, 3.00 mg; vitaminB2, 9.60 mg; vitamin B12, 0.03 mg; biotin, 0.15 mg; folic acid, 1.50 mg; pantothenic acid, 13.80 mg; nicotinic acid, 45 mg.

[2] The trace mineral premix provided (per kilogram of diets) the following: Cu, 16 mg; Zn, 110 mg; Fe, 80 mg; Mn, 120 mg; Se, 0.30 mg; I, 1.50 mg.

[3] Design values of nutrient components.
